# Extensive gene flow in secondary sympatry after allopatric speciation

**DOI:** 10.1093/nsr/nwac280

**Published:** 2022-12-12

**Authors:** Xinfeng Wang, Ziwen He, Zixiao Guo, Ming Yang, Shaohua Xu, Qipian Chen, Shao Shao, Sen Li, Cairong Zhong, Norman C Duke, Suhua Shi

**Affiliations:** State Key Laboratory of Biocontrol, Guangdong Key Laboratory of Plant Resources, School of Life Sciences, Southern Marine Science and Engineering Guangdong Laboratory (Zhuhai), Sun Yat-sen University, Guangzhou510275, China; Ministry of Education Key Laboratory for Biodiversity Science and Ecological Engineering, School of Life Sciences, Fudan University, Shanghai200438, China; State Key Laboratory of Biocontrol, Guangdong Key Laboratory of Plant Resources, School of Life Sciences, Southern Marine Science and Engineering Guangdong Laboratory (Zhuhai), Sun Yat-sen University, Guangzhou510275, China; State Key Laboratory of Biocontrol, Guangdong Key Laboratory of Plant Resources, School of Life Sciences, Southern Marine Science and Engineering Guangdong Laboratory (Zhuhai), Sun Yat-sen University, Guangzhou510275, China; Department of Laboratory Medicine & Pathology, University of Washington, Seattle, WA98195, USA; State Key Laboratory of Biocontrol, Guangdong Key Laboratory of Plant Resources, School of Life Sciences, Southern Marine Science and Engineering Guangdong Laboratory (Zhuhai), Sun Yat-sen University, Guangzhou510275, China; State Key Laboratory of Biocontrol, Guangdong Key Laboratory of Plant Resources, School of Life Sciences, Southern Marine Science and Engineering Guangdong Laboratory (Zhuhai), Sun Yat-sen University, Guangzhou510275, China; State Key Laboratory of Biocontrol, Guangdong Key Laboratory of Plant Resources, School of Life Sciences, Southern Marine Science and Engineering Guangdong Laboratory (Zhuhai), Sun Yat-sen University, Guangzhou510275, China; State Key Laboratory of Biocontrol, Guangdong Key Laboratory of Plant Resources, School of Life Sciences, Southern Marine Science and Engineering Guangdong Laboratory (Zhuhai), Sun Yat-sen University, Guangzhou510275, China; Hainan Academy of Forestry (Hainan Academy of Mangrove), Haikou571100, China; Centre for Tropical Water and Aquatic Ecosystem Research, James Cook University, Townsville, QLD 4811, Australia; State Key Laboratory of Biocontrol, Guangdong Key Laboratory of Plant Resources, School of Life Sciences, Southern Marine Science and Engineering Guangdong Laboratory (Zhuhai), Sun Yat-sen University, Guangzhou510275, China

**Keywords:** mangroves, population genomics, speciation, introgression, species hybridization

## Abstract

In the conventional view, species are separate gene pools delineated by reproductive isolation (RI). In an alternative view, species may also be delineated by a small set of ‘speciation genes’ without full RI, a view that has gained broad acceptance. A recent survey, however, suggested that the extensive literature on ‘speciation with gene flow’ is mostly (if not all) about exchanges in the early stages of speciation. There is no definitive evidence that the observed gene flow actually happened after speciation is completed. Here, we wish to know whether ‘good species’ (defined by the ‘secondary sympatry’ test) do continue to exchange genes and, importantly, under what conditions such exchanges can be observed. *De novo* whole-genome assembly and re-sequencing of individuals across the range of two closely related mangrove species (*Rhizophora mucronata* and *R. stylosa*) reveal the genomes to be well delineated in allopatry. They became sympatric in northeastern Australia but remain distinct species. Nevertheless, their genomes harbor ∼4000–10 000 introgression blocks averaging only about 3–4 Kb. These fine-grained introgressions indicate continual gene flow long after speciation as non-introgressable ‘genomic islets,’ ∼1.4 Kb in size, often harbor diverging genes of flower or gamete development. The fine-grained introgression in secondary sympatry may help settle the debate about sympatric vs. micro-allopatric speciation. In conclusion, true ‘good species’ may often continue to exchange genes but the opportunity for detection is highly constrained.

## INTRODUCTION

Biological species are generally defined as taxa that do not exchange genes due to various forms of reproductive isolation (RI). RI mechanisms include ecological, behavioral, and reproductive incompatibilities [1,2]. These mechanisms are the foundation of the Biological Species Concept (BSC) [3–5] and have been accepted as both necessary and sufficient for species to evolve along diverging paths.

Strictly speaking, two true species should be separated by a combination of RI mechanisms such that they cease to exchange genes anywhere in their genomes. RI needs to be complete to avoid the logical quagmire of defining how much isolation is enough. In addition, RI assumes a very high degree of genetic cohesiveness within each species such that any exchange would be harmful [3,5,6]. Complete RI, however, is difficult to ascertain. For example, many good species appear to be only partially isolated as they produce sterile hybrids in one sex but the other sex is highly fertile [7–11]. In many other cases, there is no evidence even for partial RI [12–15]. An equally thorny question is the many seemingly convincing cases of sympatric speciation, say, between fish species in crater lakes [16–18]. A common defense of BSC in such cases is that it is often micro-allopatric speciation imposing restricted gene flow in a small spatial scale.

Against this backdrop, the basic assumptions of BSC in asserting full RI across the whole genome has been questioned. In reality, only a small fraction of the genome may be responsible for differentiation between species in morphology, behavior, reproduction, and ecology (i.e. ‘speciation genes’). In this genic view of speciation (GVS) [5], genome regions not germane to speciation should be easily interchangeable between species. In short, BSC asserts that true species should be fully reproductively isolated. The alternative GVS, by allowing species to extensively share genetic material, would abandon RI as the defining concept of species. The central question is, therefore, ‘do true species extensively exchange genes?’

### Conditions for detecting post-speciation gene flow and the literature on speciation with gene flow

While there is an extensive literature on gene flow during, or even after, speciation (reviewed in [6]), a series of recent debates raise the possibility that the evidence is almost entirely about gene flow in the early phase of speciation [6,19–23]. In other words, there is still no definitive evidence that gene flow continues through the entire process of speciation and extends to ‘good species’.

**Figure 1.**
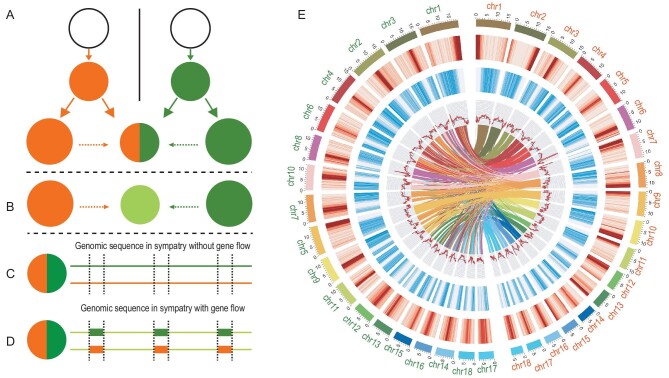
Models of post-speciation gene flow and the genomic structure in such models. (A) Two populations undergo allopatric speciation, resulting in two good species depicted in orange and green, respectively. In an area away from the center of each species’ distribution, the two putative species re-establish sympatry. In sympatry, the two species retain their biological characters (shown in their distinctive colors), passing the ‘secondary sympatry’ test. (B) In many cases, the two putative species hybridize and fuse (indicated by the olive color) in sympatry. They are not considered true species. (C and D) When the two species pass the sympatry test, there are two possibilities. In (C), the two species do not exchange genes at all. This is the most common scenario as observed in many secondary sympatry cases. (In such cases, the hybrid zones may show some admixture although the hybrids are usually quickly eliminated by natural selection.) (D) The true case of post-speciation gene flow. The genomic sequences of the two species show signs of genetic exchanges in the genomic segments of olive color. The small genomic segments flanked by dotted lines are non-introgressable and may harbor ‘speciation genes.’ It should be noted that the patterns of introgression are transient. As the two species continue to diverge, gene flow within the same species will homogenize the genomic sequences of each species, thus reversing the genomic patterns back to that shown in (C). (E) The genomes of *R. mucronata* and *R. stylosa*. Circular tracks represent, from outer to inner, top 18 longest scaffolds of *R. mucronata* (chr1–18 in orange) and *R. stylosa* (chr1–18 in green), percentage of repeats (3.97–99.92%, the darker the higher), gene density (0–47, the darker the higher), GC content (29.73–51.97%) and the spectrum of inter-specific collinear analysis (each line connects one pair of homologous genes and a cluster of such lines represents one collinear block). All statistics are calculated for windows of 200 Kb.

We may use the model outlined in Fig. 1A–D to evaluate the conditions for detecting post-speciation gene flow. To begin with, the species status needs to be defined by unambiguous criteria. The most stringent of them are: First, two populations have evolved into putatively full species in allopatry. Second, the two species subsequently come into sympatry but maintain their biological distinction over an extended period, even with ample opportunities for genetic exchanges to merge into a hybrid swarm (Fig. 1A). These criteria are the classical and most stringent definition of species [1,4,5,11] which shall be referred to simply as the ‘secondary sympatry’ test in this study. The sympatry test is important as seen in previous debates on this issue [6,24–26]. In many cases, the species may not pass the sympatry test as shown in Fig. 1B (see the legends). Furthermore, while the extensive genomic literature has convincingly demonstrated gene flow during speciation [6], the inferred gene flow most likely happened in the early stages. Note that the focus here is on post-speciation gene flow.

Once good species are confirmed by the test, it would be possible to check for past genetic exchanges discernible in their genomes. Importantly, the extensive survey by Wang *et al.* finds no convincing evidence for post-speciation gene flow [6]. Such evidence may not be readily accessible as the window of time for convincing observations is quite narrow. If it is too early, the species may not pass the ‘secondary sympatry’ test (Fig. 1B). On the other hand, the footprint of post-speciation gene flow may not last long as subsequent divergence and gene flow would erase it (Fig. 1C).

The true signature of post-speciation gene flow requires two signals. First, allopatric populations of these species should show the pattern depicted in Fig. 1C. Second, sympatric populations would show the Fig. 1D pattern. The contrast between the two signals ensures that the gene flow in sympatry has indeed happened after speciation has completed. As stated above, gene flow during speciation, as concluded in many studies [6,27,28], most likely happens in the early stages. The model illustrated in Fig. 1A–D shows the challenges of proving post-speciation gene flow. A convincing proof, requiring careful experimental design to identify species at the right stage and in the right place of evolution, would be highly conceptually significant. This study provides such a proof by sequencing a large number of genomes of two closely-related mangrove species *Rhizophora mucronata* and *R. stylosa.* These samples are collected from 11 populations across their range of distribution over a large geographical area.

Mangroves are woody plants that have colonized intertidal zones of tropical coasts [29–32]. *R. mucronata* and *R. stylosa* are distributed on the Indo-Western Pacific (IWP) coasts [31]. They have distinct but overlapping geographical distributions and have diverged consistently in morphological and ecological characteristics such as the style length and saline tolerance [31,33]. Because of the narrow band of suitable habitats along the coasts (or near river mouths), global mangrove distributions are essentially one-dimensional, making them ideal for biogeographical studies of speciation.

## RESULTS

### *De novo* assembling *R. Mucronata* and *R. Stylosa* genomes

We sequenced the genome of one *R. mucronata* and one *R. stylosa* individual, respectively (Supplementary Table S1; see Supplementary Table S2 for details). Although the genomes of these species have been re-sequenced before [30], the aims of this study demand accurate assembly of their own genomes in order to assess possible fine-grained introgressions. We therefore carried out *de novo* sequencing and attained the chromosome-level assembly in this study. These two genomes were sequenced as a part of the Genomics of World Mangroves Project as described in several of our previous publications [34,35], in particular, the phylogenomic analysis [36]. The full description of genome sequencing, annotation and analyses are provided here, upon which the re-sequencing of individuals from multiple populations is based.

We obtained 33.84 Gb (gigabases) and 36.13 Gb of raw data, corresponding to 142X–164X coverage of the assembled genomes (Supplementary Table S2). We assembled 237.85 Mb (megabases) of *R. mucronata* sequences into 14 496 scaffolds with N50 at 12.03 Mb and 18 scaffolds (chr1–18) >= 5 Mb. The assembled chr1–18 account for 84.38% (200.70 Mb) of the genome and correspond to the diploid chromosome number (2n = 36, Fig. 1E, Supplementary Table S1 and Fig. S1). The assembled *R. stylosa* genome is 219.67 Mb, containing 319 scaffolds with N50 at 12.64 Mb. The top 18 scaffolds account for 218.34 Mb, or 99.39%, of the genome (Fig. 1E, Supplementary Table S1 and Fig. S2). The sizes of our assemblies were smaller than the estimated genome sizes by flow cytometry and k-mer analysis, which range from 352.03 to 369 Mb in *R. mucronata* and from 299.71 to 307 in *R. stylosa* [36]. We were able to map 95.2% and 95.9% of the 2326 BUSCOs, as well as 94.52% and 92.53% of the corresponding Illumina sequencing reads, to the genome assemblies of *R. mucronata* and *R. stylosa*, respectively (Supplementary Tables S1 and S2). These results indicate the high completeness of our *de novo* assembled genomes, approaching a chromosomal level. A total of 22 574 gene models were annotated and classified into 17 897 families in the *R. mucronata* genome, while 23 545 genes from 18 134 families were identified in *R. stylosa* (Supplementary Table S1). This gene prediction comes also with high completeness, indicated by the fact that 89.5% and 91.4% of the 2326 BUSCOs were successfully mapped to our predicted gene models of *R. mucronata* and *R. stylosa*, respectively (Supplementary Table S1).

To examine the evolution of *R. mucronata* and *R. stylosa* genome structure in the larger phylogenetic context, we compare their genomes to those of *Carallia pectinifolia* [36], *Bruguiera gymnorrhiza* [32] and *Rhizophora apiculata* [30]. *C. pectinifolia* is from one of the closest non-mangrove genera in the Rhizophoraceae family; 29 653 gene families were identified among the five species, with 6890 single-copy. Using these single-copy orthologs, we reconstructed the species’ phylogeny and estimated divergence times (Supplementary Fig. S3). The phylogeny shows that *R. mucronata* and *R. stylosa* are sisters to each other diverging ∼2.70 Mya (with 95% confidence interval [2.05, 3.44] Mya; Supplementary Table S3 and Fig. S3). *R. mucronata* and *R. stylosa* are likely to be most closely related to each other as shown previously [36]. Most important, they meet the requirements for detection of post-speciation gene flow within a fairly small window of time after speciation (see Discussion). We identify 837 collinear blocks between the two species that harbor 18 716 genes in *R. mucronata* and 18 663 in *R. stylosa* (Fig. 1E). The inter-specific Ks (∼0.0031) and the genomic divergence (*D_xy_* = 0.0031) are relatively low (Supplementary Table S2 and Fig. S4).

### *R. Mucronata* and *R. Stylosa* genomic diversity

**Figure 2.**
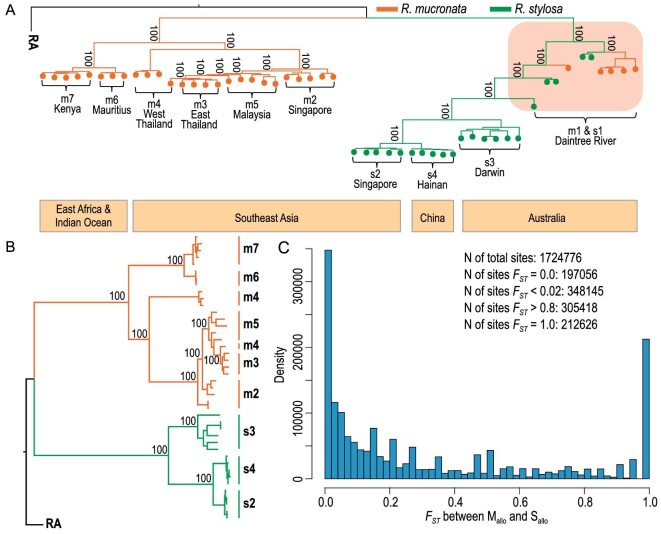
Phylogenetic relationships and biogeographical observations. (A) The genealogy of *R. mucronata* and *R. stylosa*. The maximum-likelihood (ML) tree, generated by RAxML with 100 bootstraps. The numbers on the nodes indicate support values. *R. mucronata* is colored in orange while *R. stylosa* is in green. Orange boxes below the phylogeny indicate geographical regions of the sampled populations (see Supplementary Table S4 for details). The sympatric *R. mucronata* (m1) and *R. stylosa* (s1) in Daintree River, Australia are highlighted with red background. (B) The same phylogeny excluding the sympatric m1 and s1 samples from the Daintree area. We denote the allopatric populations as M_allo_ (m2–m7) and S_allo_ (s2–s4). (C) The spectrum of the *F_ST_* statistic between the M_allo_ and S_allo_ samples.

*Rhizophora mucronata* is widely distributed in the Indo-Western Pacific (IWP) region, particularly to the west of the Strait of Malacca and all the way to East Africa. In contrast, *R. stylosa* extends eastward from the Strait of Malacca to western Pacific islands (Fig. 2A). The two species have been reported to overlap in scattered locales along several western Pacific coastlines. However, in our own field trips, their relative abundance is often skewed in favor of one species and their co-occurrence has rarely been found. The sole exception in our collection is in the Daintree River (DR) area of northeastern Australia, where both species are quite abundant (Fig. 2A).

We collected 21 *R. stylosa* individuals from four locations (labeled s1–s4) and 31 *R. mucronata* samples from seven locations (m1–m7) for population genomic studies (Fig. 2A and Supplementary Table S4). Note that m1 and s1 refer to the sympatric DR samples. Whole genomes of all samples were sequenced on the Illumina Hiseq 2000 platform, yielding a mean depth of 16X (ranging from 12X to 22X) (Supplementary Tables S5 and S6). Short reads from each individual were mapped to the *de novo R. mucronata* genome, with average genomic coverage of 81% (80%–83%, Supplementary Table S4). The level of genetic diversity shows two patterns. Low genetic diversity is found in all allopatric populations (average θ_π_ at 0.62 and 0.60 per Kb for *R. mucronata* m2–m7 and *R. stylosa* s2–s4, respectively, see also Supplementary Tables S2 and S4). The level is much higher in the sympatric DR populations (θ_π_ = 1.37/Kb and 2.09/Kb, respectively). Watterson's estimates (θ_w_) are similar (Supplementary Table S4, see Supplementary Methods).

### Divergence between the two species in allopatry

Genomic divergence (*D_xy_*) between the two species is 4.14 × 10^−3^ per site (Supplementary Table S7). We first constructed a Maximum Likelihood (ML) tree using RAXML [37] on 31 *R. mucronata* and 21 *R. stylosa* individual sequences from the 11 populations. The ML tree bifurcates with a clear delineation between species across all allopatric populations. However, the m1 and s1 (i.e. DR) samples show strong signs of admixture as they are ‘in the middle’ of the bifurcated tree (Fig. 2A). When the DR samples are removed, the phylogeny shows clear delineation (Fig. 2B). These two trees are robust when rebuilt using the ML method in IQTREE [38] or the Neighbor-Joining (NJ) method in MEGA7 [39] (Supplementary Figs. S5–S6). The monophyletic delineation of *R. mucronata* and *R. stylosa* in allopatry is also supported by a principal component analysis (PCA, Supplementary Fig. S7a) [40].

We detected 1.7 million variable sites across all populations of the two species (Supplementary Table S8). We first partition these sites by excluding the DR samples (see Supplementary Methods). Each site is then represented by an *F_ST_* value with *F_ST_* = 0 indicating no differentiation between the two species in allopatry and *F_ST_* = 1 indicating complete differentiation. Figure 2C shows the U-shaped distribution where the abundance of sites at the far right reveals the extensive differentiation between species. At the other end, the sites with low differentiation may indicate gene flow between species. Such a U-shape distribution is typical of species diverging with little gene flow [41]. In contrast, the distribution between *R. mucronata* and *R. stylosa* samples in the DR region (m1 vs. s1) is the typical L shape, suggesting extensive introgression within this region (Supplementary Fig. S7b).

**Figure 3.**
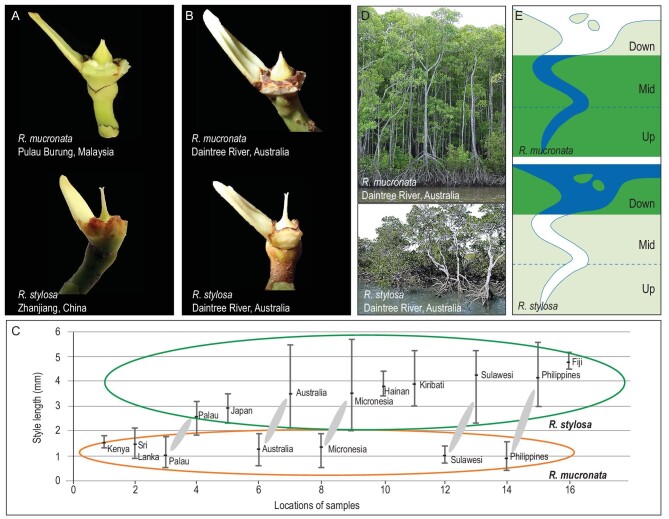
Key diagnostic characters between *R. mucronata* and *R. stylosa*. (A) *R. mucronata* (Pulau Burung, Malaysia, 101°50'14.6''E, 2°29'33.7'N) and *R. stylosa* (Zhanjiang, China, 109°45'46.50''E, 21°34'7.32''N) styles from allopatric sites. (B) *R. mucronata* and *R. stylosa* styles from the Daintree River, Australia, sympatric site. (C) Variation of style lengths of *R. stylosa* (green oval) and *R. mucronata* (orange oval) throughout the Indo-West Pacific region. Solid ellipses contrast sympatric samples. (D) General tree morphology of *R. mucronata* and *R. stylosa* from the Daintree River, Australia, sympatric site. (E) Diagrams showing the habitat preferences of *R. mucronata* and *R. stylosa* in a typical estuary (adapted from mangrove ID [75]).

The two species can be easily distinguished in the field. *R. mucronata* tends to settle in the less saline and further upstream habitats in comparison with *R. stylosa* found in saline habitats closer to river mouths (see Fig. 3E). The two species also differ substantially in overall tree morphology (see Fig. 3D). They are most readily distinguished by the reproductive characters of the flower, in particular the style length [31,33] as pictured in Fig. 3A. The morphological differences between *R. mucronata* and *R. stylosa* across populations are shown in Fig. 3C. *R. mucronata* is readily distinguished by its short style, between 0.9 and 1.6 mm (Fig. 3A). In contrast, the *R. stylosa* style is long, 2.4–5.3 mm (Fig. 3A and C) [31,33]. While the style length varies from locale to locale in both species, this trait is species-diagnostic across locales. Additional, albeit less stable, diagnostic morphological characters are listed in the Supplement (Table S9). In short, we show that *R. mucronata* and *R. stylosa* have diverged in their genomes, geographical distribution, habitat choice, and various morphological characters.

### Characterizations of *R. Mucronata* and *R. Stylosa* in sympatry

We next apply the ‘secondary sympatry’ test to these two species found at the DR site of northern Australia. DR is at the periphery of the distribution of either species (Fig. 2) with *R. mucronata* to the west and *R. stylosa* to the north. It appears that speciation between them had been completed in allopatry and the post-speciation contact happened in DR (see also Supplementary Notes). Importantly, the two species have remained distinct in their ecology and morphology for a substantial period without intermingling. The two extant species rarely produce F1 hybrids and morphological intermediates are uncommon in our field work. In particular, as we observed in the field trips, the style lengths of DR samples are concordant with that of the allopatric populations of the same species (Figure  3B and C). In the DR area, these two species are parapatric-sympatric with distributions up- or down-river and extensive overlap in the middle (Fig. 3E). This difference in habitat preference is a hallmark of the speciation between *R. mucronata* and *R. stylosa*.

**Figure 4.**
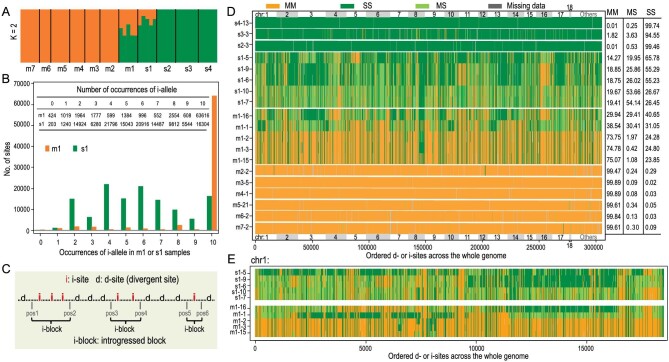
Interspersion between introgressions and non-introgressions. (A) Genetic clustering of all 52 individuals of the two species by ADMIXTURE (K = 2). Orange and green colors denote the *R. mucronata* (m) and *R. stylosa* (s) components, respectively. Individuals from each population are grouped between adjacent black lines. (B) Distribution of i-allele occurrences in m1 (orange) and s1 (green) populations. Given the five individuals (or 10 haploid genomes) from each population, the occurrence ranges from 0 to 10. Exact numbers are given in the inset table (see also Supplementary Fig. S10). (C) A schematic diagram for delineating i-blocks, each harboring consecutive i-sites without being interrupted by d-sites. The length of an i-block is determined by the midpoints of the flanking (d and i) intervals. Three i-blocks are shown. (D) The genome-wide landscape of i-blocks. Top 18 longest scaffolds (chr1–18) and the rest of the genome (others) are marked and sibling scaffolds are distinguished by gray rectangles. Each row indicates an individual with each vertical line indicating a site. All 10 individuals from the sympatric s1 and m1 populations are shown. For comparison, one individual is randomly selected from each of other populations (see Fig. S12 for the full display). Each site is color-coded for its genotype: MM (orange), MS (light green) and SS (green) type, where M stands for *R. mucronata* and S for *R. stylosa*. The percentages are summarized on the right. Note that extensive interspersions are observed only in the sympatric samples. (E) A close-up view of i-blocks in the longest scaffold (chr1). Only 10 individuals from the sympatric s1 and m1 populations are shown.

To elaborate on the phylogenetic positions of the DR samples in Fig. 2A, we used Bayesian clustering analysis implemented in ADMIXTURE [42]. We identified two genetic components that make up DR sample genomes (Fig. 4A). PCA results also indicate significant admixture in m1 and s1 individuals (Supplementary Fig. S7a). Furthermore, because species divergence is monophyletic in all allopatric comparisons, incomplete lineage sorting is an unlikely cause of the observed admixture in the DR samples (see also Supplementary Notes). In short, we interpret the high *F_ST_* sites as representing divergence after speciation (Fig. 2C) with subsequent admixture in the DR area. Additional tests of introgression (LD analysis, Patterson's *D* statistic, and the modified *f_d_* statistic) are presented in the Supplement (Tables S8 and S10 and Figs. S8 and S9).

### Extensive introgression in sympatry

Using the sympatric samples, we ask the following questions: (1) How many introgressed segments can be found in each species? (2) Is the introgression symmetric? (3) What is the introgressed segment size distribution? A few large blocks are expected if hybridization was recent but many fine-grained blocks should result from old introgressions that have been eroded by recombination. (4) How many genomic segments fail to introgress and what is their genic content? Question 4 will be the subject of the next section.

To identify DR area introgressions, we first define divergent sites (or d-sites) between *R. stylosa* and *R. mucronata* in allopatry. Among the d-sites, we can then define introgressed sites (i-sites) between the m1 and s1 samples in sympatry. There are 305 418 d-sites, defined as sites with *F_ST_* >0.8 between the two species in allopatry. Note that the bulk of d-sites (212 626) are fully divergent with *F_ST_* = 1.0 (Fig. 2C). An i-site is a locus where the introgressed allele (or i-allele) is found in >= *n* of the 10 genomes in m1 or s1 sample sets, where *n* is usually equal to 8. (Note that both m1 and s1 samples have five diploid individuals, or 10 genomes.) In general, if introgression is observed in one direction, say from *R. stylosa* to *R. mucronata* in the DR area, the same site usually does not show introgression (*n* <= 1) in the reciprocal direction from *R. mucronata* to *R. stylosa.* (In this context, a non-introgressable site, or j-site, is defined as the d-site which contains zero or only one i-allele in both m1 and s1 samples; see Materials and Methods, and Supplementary Methods. Note that a small fraction of d-sites are statistically undefinable between i- and j-sites.)

To call i-sites, we first define *n* (the number of genomes carrying the introgressed allele). It is obviously better to set *n* close to the maximum of 10 for strongly penetrant introgressions. Figure 4B shows the number of introgressions in the two directions. We set *n* = 8 for the m1 samples where the i-allele is usually found >= 8 times (orange bars in Fig. 4B). Hence, the results with *n* = 2 and *n* = 8 would not be very different. Furthermore, to avoid the confounding presence of remnant ancient polymorphisms, we require introgressions at an i-site to be strongly asymmetric: >= *n* one way (say, from *R. stylosa* to *R. mucronata*) and <= 1 in the reciprocal direction (Supplementary Fig. S10). I-allele counts are close to uniformly distributed between 2 and 10 (green bars in Fig. 4B) among *R. stylosa* (s1) samples. The asymmetry is probably due to the geography of the DR area, which is at the fringe of the *R. mucronata* distribution. Consequently, gene flow from *R. mucronata* into *R. stylosa* may be more limited here, resulting in the lower frequency of introgressions in the s1 samples. In this regard, setting *n* = 8 would miss many introgressions in *R. stylosa* leading to a much lower introgression rate than in *R. mucronata*. Nevertheless, the final estimates appear robust even when *n* is set as low as 2 (see below). Simulations of these scenarios are presented in the Supplement.

Introgression necessarily starts with the exchange of large genomic segments, but subsequent recombinations would gradually erode the introgressed segments into smaller fragments, resulting in short segments of DNA consisting of consecutive i-sites. We call these segments ‘introgression blocks’ (or i-blocks; Supplementary Fig. S11). Figure 4C shows a segment of the genome that comprises a string of d-sites and i-sites as defined above. These d- and i-sites are embedded in a background of low-*F_ST_* or invariant sites shown as dots. This figure shows three i-blocks, each consisting of one, two, or three i-sites. The length of each block is defined by the distance between the two breakpoints flanking it. Unless otherwise specified, we remove the singleton i-blocks that harbor only a single i-site when presenting i-block length distributions.

The analysis of i-blocks is summarized in Table 1 (see also Supplementary Tables S11–S13 and Supplementary file2). We focus on the results with *n* = 8 but the results with *n* = 2 and *n* = 10 are given for comparison. In the DR area, *R. mucronata* (m1) samples harbor far more introgressions than *R. stylosa* (s1). The bottom of Table 1 at *n* = 8 shows that 16.09 or 23.09% of the *R. mucronata* genomes are introgressions from *R. stylosa*, the two values depending on whether singleton i-blocks are counted. In the opposite direction, 7.97–12.06% of the *R. stylosa* genomes are introgressions. The introgressions in Table 1 are visualized in Fig. 4. The salient observation is the highly fine-grained nature of the introgressions (Fig. 4 and Supplementary Figs. S12–S13). In *R. mucronata*, the introgressions are distributed over 9963 i-blocks with an average length of 3.24 Kb (Table 1 and Supplementary file2). In *R. stylosa*, there are 3874 i-blocks with an average size of 4.13 Kb (Table 1 and Supplementary file2). Thus, there likely were numerous recombination events that broke introgressions into thousands of tiny i-blocks. It should be noted that Table 1 and Fig. 4 present only the extreme cases of introgressions that rise to very high frequencies.

**Table 1.** Summary of high-penetrance introgressed i-blocks between sympatric species.

	>= 2 occurrences of i-allele		>= 8 occurrences of i-allele		=10 occurrences of i-allele	
	m1	s1	m1	s1	m1	s1
**No. of i-blocks (No.** **of** **scaffolds with i-blocks)**	9742 (18)	8228 (18)	9963 (18)	3874 (18)	9741 (18)	2219 (18)
**Length of i-block—Range (mean)**	5 bp −944.95 Kb (3.14 Kb)	8 bp–938.88 Kb (3.49 Kb)	5 bp–944.95 Kb (3.24 Kb)	8 bp–938.88 Kb (4.13 Kb)	5 bp–944.95 Kb (3.02 Kb)	8 bp–137.48 Kb (4.15 Kb)
**No. of i-sites in a block—Range (total i-sites)**	2–356 (40 780)	2–116 (42 862)	2–349 (42 572)	2–116 (19 958)	2–132 (40 727)	2–100 (14 868)
**Total length of i-blocks (% of the genome)**	30.63 Mb (15.26%)	28.73 Mb (14.31%)	32.29 Mb (16.09%)	16.00 Mb (7.97%)	29.54 Mb (14.72%)	9.22 Mb (4.59%)
**Total length of i-blocks** ^a^ **(% of the genome)**	45.33 Mb (22.58%)	39.61 Mb (19.74%)	46.33 Mb (23.09%)	24.21 Mb (12.06%)	43.14 Mb (21.49%)	15.57 Mb (7.76%)

Note that the species origin of introgressed alleles (i-alleles) is first defined in the allopatric populations. Hence, i-alleles in the DR area can be identified even when they are bi-directional. All i-alleles in this table are uni-directional with, for example, > = 8 in one direction, while the reciprocal direction has < = 1 i-allele. An introgressed block (i-block), unless explicitly stated, has > = 2 introgressed sites (i-sites). The 18 scaffolds collectively account for 84.38% (200.70 Mb) of the whole genome (237.85 Mb). ^a^These include singleton i-blocks.

The distributions of i-blocks are shown at the large genomic scale in Fig. 4D, at the scaffold scale in Fig. 4E and as individual sites in Supplementary Fig. S13A–S13C. Note that only d-sites and i-sites are displayed in these figures. As shown in Fig. 2C, the d- and i-sites are the 305 418 sites with *F_ST_* > 0.8. The rest are invariant or close to invariant sites. The i-blocks are dispersed across the whole genome (Fig. 4D and Supplementary Fig. S12). Indeed, all top 18 scaffolds harbor numerous transitions between i- and d-blocks both in m1 and s1 genomes (Fig. 4D and Table 1). Figure 4E shows that transitions between i- and d-blocks can occur in a few to tens of Kb. At the site level, i-blocks and d-blocks can switch within a small distance (Supplementary Fig. S13A–S13C). An i-block (or d-block) may harbor only one i-site (or d-site), referred to as a singleton block (Table 1, Supplementary Table S11 and Fig. S13). Singleton blocks, not uncommon but less reliable, are not used in the tally.

The extensive fine-grained introgressions convey two messages. First, hybridizations may happen continually over a long period of time. Each hybridization event would initially bring in whole-chromosome introgressions that are subsequently broken down by recombination. Small DNA fragments may have been continually introgressed in this piecemeal manner. Second, loci underlying differential adaptation between species may be very common such that introgressions tend to be small, and thus free of the introgressed alleles that are deleterious in the genetic background of another species [43]. In the next section, we will direct our attention toward non-introgressions, which are blocks of native alleles flanked by introgressed DNA segments.

### Very fine-grained interspersion between ‘introgressable’ and ‘non-introgressable’ blocks

Some DNA segments may not be introgressable due to the presence of adaptively significant genes. Such loci, by definition, contribute to reproductive isolation or ecological speciation [5,44] and have sometimes been referred to as ‘speciation genes’ [11,45–50]. The number, size, and direction of introgressions, therefore, depend on several parameters: (1) hybridization rate; (2) the strength of selection against the speciation genes when introgressed; (3) the number and location of speciation loci; (4) recombination rate; and (5) the length of time since initial hybridization.

To test the evolutionary forces shaping the genomic pattern of introgression, we carried out computer simulations based on the Recurrent Selection and Backcross (RSB) model [51] in a companion study [6] (see also Materials and Methods, and Supplementary Methods). The RSB model has been proposed for identifying genes’ underlying complex traits [51]. It involves repeated dilution of the genome of breed A (say, the bulldog) with that of breed B (e.g. the Border Collie) while retaining the desired phenotypic traits of the former. This is done by continually selecting for the traits of breed A while backcrossing to breed B. The scheme is almost identical to the process of ‘speciation with gene flow.’ They differ only in the parameter values; for example, the length of time in speciation is far greater and gene flow is much smaller, and often bidirectional. The differences necessitate separate simulations for speciation with gene flow. As shown in Fig. 2 of Wang et al. [6] and Supplementary Figs. S13 and S14 in this study (see also Supplementary Methods and Fig. S14), introgressions are fine-grained around almost all speciation genes. These patterns resemble the observations reported in this study.

Consistent with the observation from computer simulations, we indeed observed a number of non-introgressable genome blocks in *R. mucronata* and *R. stylosa*. With the j-site defined above, a j-block (i.e. non-introgressable block) is defined as a DNA segment containing at least one j-site (Table 2). Using these stringent criteria, we see ∼1200 j-blocks which together account for <1% of the genome and harbor 328 coding genes of which 171 contain j-sites (Table 2, Supplementary Table S14 and Fig. S15). For higher confidence, we also show j-blocks with at least two j-sites (Table 2). While only 19 genes containing j-sites are found in these j-blocks (Supplementary Table S15), it is remarkable that six of the 19 genes function in flower development and/or gamete production and development as shown in Table 2 (see the WEGO gene ontology in Supplementary Fig. S15, where a larger set of genes is presented under less stringent criteria). One (*RM_77078.7*) of the six genes, known as *EMF1* (embryonic flower 1), regulates reproductive development and is involved in controlling flowering development [52,53]. *RM_76773.10, RM_76979.9* (*NAC2*) and *RM_77530.24* are involved in regulating the stamen development, pollen germination and tube growth [54–56]. *RM_76929.10, RM_76979* and *RM_77333.68* all play a role in embryonic development [57–59]. Since all six genes contain highly differentiated amino acids and non-introgressable sites (j-sites) (Table 2, Supplementary Table S15 and Fig. S16), their involvement in speciation between *R. mucronata* and *R. stylosa* seems plausible.

**Table 2.** High-confidence non-introgressable j-blocks for the identification of genes involved in speciation.

				> = 1 j-sites	> = 2 j-sites
No. of j-blocks (No. scaffolds with j-blocks)				1189 (184)	168 (44)
Length of j-blocks—Range (mean)				3 bp–43.51 Kb (1 010 bp)	23 bp–35.75 Kb (1 062 bp)
No. of j-sites in a block—Range (total j-sites)				1–6 (1443)	2–6 (422)
Total length of j-blocks (% of the genome)				1 201 823 bp (0.51%)	178 520 bp (0.075%)
No. of genes with j-sites				171	19
No. of genes of flower development with j-sites				–	6 (see below)
**Gene name**	**j-sites**	**L(aa)**	**Site** **^a^**	**Function in *Arabidopsis thaliana***	
***RM_76773.10*** (*AT2G14110*)	3	255	4	Haloacid dehalogenase-like hydrolase (HAD) superfamily protein. Participating in pollen germination and tube growth [54].	
***RM_76929.10*** (*AT1G55490*)	2	294	3	CPN60B. Mutants in this gene develops lesions on its leaves and accelerated cell death due to heat shock stress. Participating in embryo and seed development [57].	
***RM_76979.9*** (*AT3G15510*)	2	199	1	NAC2 (NAC domain containing protein 2). Involved in the regulation of stamen development and embryonic development [55,58].	
***RM_77078.7*** (*AT5G11530*)	3	1415	1	EMF1 (embryonic flower 1). Involved in regulating reproductive development [52,53].	
***RM_77333.68*** (*AT1G08520*)	2	755	1	ALB1. Encoding the CHLD subunit of the Mg-chelatase enzyme which is involved in chlorophyll biosynthesis. Participating in embryo and seed development [59].	
***RM_77530.24*** (*AT3G05420*)	2	671	2	ACBP4 (acyl-CoA binding protein 4). Expressed and function in floral lipid metabolism. Playing combinatory roles in pollen development [56].	

A j-block, unless explicitly stated, has > = 2 non-introgressable sites (j-sites). j-sites: the number of non-introgressable sites within the gene. L(aa): amino acid sequence length of the gene. ^a^Site: No. of highly differentiated amino acids between *R. mucronata* and *R. stylosa* are given (see Supplementary Fig. S16).

## DISCUSSION

*R. mucronata* and *R. stylosa* in the DR area affirm their species status as they retain their biological characteristics in sympatry. However, we found numerous introgressions where the two species coexist. Since these exchanged segments are small on average, we infer that post-speciation gene flow may have lasted for a long time. There appear to be few exchanges at present with rare F1 hybrids found in DR, as well as at other sampling sites. For example, the m2/s2 collections from Singapore show the expected phylogenetic relationship of their species designation (Fig. 2A and B). Low hybridizations at present have also been reported in Brandan, Indonesia [60]; Panay Island, Philippines; Kosrae, Micronesia; Yap, Micronesia; and North Sulawesi, Indonesia [61].

*R. mucronata* and *R. stylosa* appear to have come into sympatry in the DR area at the right time for extensive post-speciation gene flow to occur. Northern Australia has been suggested as the place where the two species first came into contact [31]. In this interpretation, the two diverging taxa moved eastward either along the northern coasts of the Indian Ocean to Southeast Asia or by crossing the Indian Ocean to Australia [31]. *R. mucronata* in Southeast Asia then dispersed south and eastward to Australia, while *R. stylosa* in Australia migrated further east and north into SE Asia (see also Supplementary Notes) [31].

The fine-grained introgressions we find in *R. mucronata* and *R. stylosa* suggest that several conditions during these species’ evolution. First, the diverging populations must have been at the right evolutionary stage when they first came into contact. Had the secondary contact happened earlier, the process of speciation could have been arrested or reversed. Conversely, if the contact would have happened too late, there would be too little gene flow to achieve extensive introgression. The second condition may be even more difficult to satisfy: the two species had to remain in secondary contact for a long time [4]. This is because numerous recombination events, accumulated over a long period, are necessary to achieve the fine-grained introgression. The third condition is ecological. Sympatric species without niche separation would face competitive exclusion [62]. *R. mucronata* and *R. stylosa* had evolved a degree of niche separation that results in incomplete overlap in habitat preference (Fig. 3E).

The fine-grained pattern of introgression between *R. mucronata* and *R. stylosa* we find is exceptional in the literature. Nevertheless, the rarity of such observations does not necessarily mean that post-speciation gene flow is unusual. It only means that the opportunities for such observations may be rare. Indeed, *R. mucronata* and *R. stylosa* in the DR area represent the confluence of the three conditions presented above. Post-speciation gene flow could not be easily detected without careful planning.

The extensive gene flow in secondary sympatry also appears to remove the main doubts about sympatric speciation which permits nearly unimpeded gene flow. However, in the genic view of speciation, the homogenizing effect of gene flow is thwarted by diverging selective pressures between two adjacent environments. This force opposing the homogenization is not operative in sympatry as there is only one shared environment. Hence, the fine-grained introgressions observed in secondary sympatry here can shed light on the nature of sympatric speciation. A recent study addresses this issue of micro-parapatry vs. sympatry between two sub-species of fishes that evolved divergently in a fully isolated crater lake.

In this study, we interpret BSC to require complete RI as proposed in the original literature [3] (see [5] for further analyses). Although many have argued that BSC should tolerate ‘a little’ gene flow (textbooks like Futuyma's) [1], the argument is conceptually inconsistent with BSC. It also makes RI an operationally undefinable quantity. After all, genomic studies have suggested that more than a third of the genome may be exchangeable between species [5,6,11,25,63]. In conclusion, BSC and the full RI should be abandoned as a key criterion for species delineation given the many recent genomic studies [11,29,43,64–68] and the observations reported here.

## MATERIALS AND METHODS

### Plant material, genome sequencing and assembly

*R. mucronata* and *R. stylosa* individuals were collected for whole-genome sequencing from Dongzhai Harbor, Hainan, China (110°35'5.79'' E, 19°56'39.67'' N, Supplementary Tables S1 and S2), although the *R. mucronata* individual was originally introduced from Australia [69]. Genomic DNA extraction from leaves was performed following the CTAB method [70]. Total RNA was extracted from leaves, flowers, and fruits using the modified CTAB method [71]. Short-read libraries were constructed and sequenced using the BGISEQ-500 platform; 50 Kb long-read libraries were prepared using the 10X Genomics (Illumina Hiseq X Ten) platform. We also re-sequenced 31 *R. mucronata* individuals from seven populations and 21 *R. stylosa* individuals from four populations using the Illumina Hiseq 2000 platform. The detailed methods of sequencing, assembling, annotation, collinearity analysis, SNP calling and divergent time estimation are given in Supplementary Methods.

### Detecting gene flow

We applied Patterson's *D* statistic and a modified *f_d_* statistic to quantify gene flow [72,73]. A positive *D* or *f_d_* value is an indicator of introgression. The basic model has three ingroups (P_1_, P_2_, and P_3_) and the outgroup (O) in the genealogical relationship (((P_1_, P_2_), P_3_), O). In our analysis, P_1_ and P_2_ are different populations from the same species *R. mucronata* (or *R. stylosa*), while P_3_ corresponds to the other species. The outgroup is *R. apiculata* [30]. Positive *D* values imply that P_2_ and P_3_ have more shared alleles than P_1_ and P_3_. The plink-1.07 [74] software package was used to estimate linkage disequilibrium (LD), represented by the *r*^2^ statistic within each population or group. LD decay was used to test for the presence of admixture events. We also calculated LD decay in sympatric populations in Singapore (s2 and m2) and allopatric *R. mucronata* and *R. stylosa* populations as controls.

### Genomic scan for introgressed and non-introgressable blocks

We then identified introgressed sites (i-sites) and non-introgressable sites (j-sites) by using the population sequencing data. To probe the influences of hybridization, selection, and recombination on genomic sequences, we further carried out computer simulations. The detailed methods of genomic scanning for introgressed (i-blocks) and non-introgressable blocks (j-blocks) and simulations are given in Supplementary Methods.

## DATA AVAILABILITY

The sequences of this study have been deposited in National Genomics Data Center (NGDC), China National Center for Bioinformation. The whole genome sequence of *R. mucronata* and *R. stylosa* have been deposited in the Genome Warehouse (https://ngdc.cncb.ac.cn/gwh) in NGDC, under accession numbers GWHBGBM00000000 and GWHBGBK00000000 with BioProject ID PRJCA001504. Genomic raw reads of *R. mucronata* and *R. stylosa* individuals have been deposited in the Genome Sequence Archive (https://ngdc.cncb.ac.cn/gsa) in NGDC, under accession number CRA002289 and CRA001688 with the same BioProject ID PRJCA001504.

## Supplementary Material

nwac280_Supplemental_Files
