# Supplementary material for: Extensive gene flow in secondary sympatry after allopatric speciation
Source: Natl Sci Rev. 2022 Dec 12;9(12):nwac280. doi: 10.1093/nsr/nwac280 (PMC9869077; doi:10.1093/nsr/nwac280)
Supplement: nwac280_Supplemental_Files [file nwac280_supplemental_files.zip › Supplement_file1_of_NSR_MS-2022-808_12-06-2022.docx]

**Supplementary information for**

**Extensive gene flow in secondary sympatry after allopatric speciation**

**Authors:** Xinfeng Wang ^a,b,^*, Ziwen He ^a,^*^,^†, Zixiao Guo ^a,^*, Ming Yang ^c^, Shaohua Xu ^a^, Qipian Chen ^a^, Shao Shao ^a^, Sen Li ^a^, Cairong Zhong ^d^, Norman C. Duke ^e^, Suhua Shi ^a,^†

**Affiliations:** ^a^ State Key Laboratory of Biocontrol, Guangdong Key Laboratory of Plant Resources, School of Life Sciences, Southern Marine Science and Engineering Guangdong Laboratory (Zhuhai), Sun Yat-sen University, Guangzhou, Guangdong 510275, China

^b^ Ministry of Education Key Laboratory for Biodiversity Science and Ecological Engineering, School of Life Sciences, Fudan University, Shanghai 200438, China

^c^ Department of Laboratory Medicine & Pathology, University of Washington, Seattle, WA 98195, USA

^d^ Hainan Academy of Forestry (Hainan Academy of Mangrove), Haikou, Hainan 571100, China

^e^ Centre for Tropical Water and Aquatic Ecosystem Research, James Cook University, Townsville, QLD 4811, Australia

* These authors contributed equally to this work

† Correspondence should be addressed to Suhua Shi ([lssssh@mail.sysu.edu.cn](mailto:lssssh@mail.sysu.edu.cn)) or Ziwen He (heziwen@mail.sysu.edu.cn)

**Supplementary information**

**This file includes:**

Supplementary Notes

Supplementary Methods

Tables S1-S15

Figures S1-S16

Supplementary References

**Supplementary Notes**

In this study, the *de novo* sequenced *Rhizophora mucronata* and *R. stylosa* individual are abbreviated as RM and RS (Table S2), respectively. *R. mucronata* populations are labeled as m1, m2, m3, m4, m5, m6, and m7, while *R. stylosa* populations as s1, s2, s3, and s4, where "m" stands for *R. mucronata* and “s” stands for *R. stylosa* (Table S4). To refer to all the *R. stylosa* or *R. mucronata* populations, we use "S_all_" or "M_all_". We use "S_allo_" to refer to the allopatric *R. stylosa* populations s2-s4 and "M_allo_" for the allopatric *R. mucronata* populations m2-m7.

***Additional introgression tests between sympatric species***

i) When comparing the two species, we identified 325 SNPs fixed in either species and 900,474 SNPs shared by both species (Table S8). To check whether incomplete lineage sorting (ILS) is the main cause of the observed admixture in the DR samples, we re-conducted the process of identifying fixed and shared SNPs several times, by removing one population of either species each time. If m1 and s1 were removed, (i.e., the DR samples), the SNP counts changed to 215,350 and 259,759 respectively. In contrast, removing allopatric populations does not affect the locus counts much, and the numbers remain around 350 and 860,000 (Table S8). This suggests that incomplete lineage sorting as not the cause of the observed admixture in the DR samples.

ii) We also observed an increased genome-wide linkage disequilibrium (LD) in m1 and s1 (Fig. S8). Hence, if the background admixture in the allopatric populations reflects ancient polymorphism (or incomplete lineage sorting), the excess admixture we observed in m1 and s1 can be reasonably attributed to introgression private to m1 and s1.

iii) We further used the ABBA-BABA (or *D*) statistic to test for the excess of shared derived alleles due to introgression [72,73]*.* A positive “*D*” or “*f_d_*” value is an indicator of introgression (gene flow). The tests with m1 and s1 as the subject branches all showed significant positive “*D*” and “*f_d_*” values (P<0.01, Table S10). In contrast, no mean *D* values significantly deviated from 0 (P>0.5) in tests without m1 or s1 (Table S10). Using 500 Kb sliding windows, we constructed distributions of *D* values across the genome. Between 97.80% and 99.76% of the windows had positive *D* values in the tests with m1 and s1 (Fig. S9). In other words, introgression was only found between m1 and s1 locally in Daintree River, where *R. stylosa* and *R. mucronata* are found together.

***Detecting highly differentiated amino acids between R. mucronata and R. stylosa***

To check whether the genes identified in j-blocks diverged in amino acid level and bore functional effect, we identified highly differentiated amino acid sites in the j-blocks, with an in-house script. We obtained protein sequences of all 52 individuals for each gene (Table 2 and Table S15). We used the following criteria to call highly differentiated amino acids: 1) the differentiated nucleotide in the codon is a non-synonymous site and with *F_ST_* > 0.8 between M_allo_ and S_allo_ samples; 2) there are no identical homozygotes between *R. mucronata* and *R. stylosa*. We found 32 such amino acids between *R. mucronata* and *R. stylosa* in the 19 genes (Table S15). 12 of these sites are in the six genes involved in flower development and/or gamete production and development (Table 2 and Fig. S16).

***The possible evolutionary trajectory and speciation of R. mucronata and R. stylosa***

These observations re-enforce the notion that species characters vary over geographic range. Accepting that, we then ask what additional circumstances would be needed for speciation to occur and how might these conditions appear for the two *Rhizophora* species in this case study?

The characters shown for the two closely related species by these genetic and morphological studies have revealed distinct patterns and traits that appear to follow an ordered series of features tending towards genetic isolation and speciation. The order of events may have followed a scenario similar to the following [31]:

1. It started with the dispersal of Asian *R. mucronata* into a vacant habitat as conditions became suitable in the west and south, but with concurrent selection favoring drift towards arid and marine traits to differentiate East African *R. mucronata.*
2. Propagules were transported due to their exceptional ability for long distance dispersal and a specialized capability for establishment and growth in marine coastal conditions in a broad range of wet and dry climatic conditions.
3. These circumstances would have transported populations south and east towards Australia (in one or more founder events that selected a subset of genotypes which at some point became *R. stylosa*).
4. The longer style of *R. stylosa* further implies some possibly greater reliance on a particular pollinator, but this has not been established.
5. Ancestral Australian *R. stylosa* migrates further east and north along northern Australian shorelines and then into SE Asia as well as the western Pacific where at some point it re-unites with ancestral Asian *R. mucronata* (still ecologically conditioned for upstream brackish estuarine locations).
6. Before this event, Asian *R. mucronata* populations would have been expanding east and south as conditions became more suitable across the region, but the proximity and size of populations would likely have prevented significant isolation events and no further speciation would have occurred.
7. The geomorphic circumstances of continental drift and their timing were likely critical in the progress of the biological events of dispersal and speciation [76].

**Supplementary Methods:**

***Genome assembly***

Short- and long- read libraries of *R. mucronata* and *R. stylosa* samples were constructed using the BGISEQ-500 and 10X Genomics platform. Before assembling, low-quality reads, adaptor sequences, N and polyA contamination were filtered out. The 10X Genomics data was used to assemble a draft genome using SUPERNOVA [77]. To calibrate and refine the assembly, we used clean short reads and Hi-C reads based on the 3D-DNA (3D DNA *de novo* genome assembly) pipeline (https://github.com/theaidenlab/3d-dna). Hi-C libraries were constructed from fresh leaves. The leaves were fixed in formaldehyde solution to get cross-linking DNA. The purified DNA was physically sheared to a length of ~400 bp. The Hi-C libraries were then constructed on the BGISEQ-500 platform. After manual check and calibration with Juicebox (https://github.com/aidenlab/Juicebox), we anchored 18 pseudo-chromosomes (chr1-18) both for *R. mucronata* (200.70 Mb, or 84.38%) and *R. stylosa* (218.34 Mb, or 99.39%) genomes, corresponding to their diploid chromosome number of 2n = 36 (Supplementary Table S1) [78,79].

Repetitive sequences in the assembled genomes were identified using homology-based and *de novo* approaches. In the homology-based prediction, RepeatMasker v. 4.0.5 [80] and Repeat-ProteinMask [8] were used to identify transposable elements (TEs) based on the known Repbase TE library [81]. In the *de novo* approach, the *de novo* repeat library was constructed by RepeatModeler pipeline [82] and LTRFinderv.1.0.6 [83]. TEs and tandem repeats were annotated using RepeatMasker v. 4.0.5 [80] and TRFv.4.04 [84], respectively. Then all annotations from the two approaches were merged (Fig. 1 and Supplementary Table S1 and Figs. S1-S2).

The homology-based, transcriptome-based and d*e novo* prediction were used to conduct annotation of gene models, with the repeats identified before masked. For the homology-based prediction, the BLAT [85] aligned results of sequence of several relative species were used in the pipeline of GeneWise [86]. In the transcriptome-based prediction, clean RNA-sequencing reads were mapped to the assembled genome using HISAT2 v2.1.0 [87]. StringTie v1.3.3b [88] was applied to assemble and annotate the transcriptomes. The RNA-seq data have been deposited in the Genome Sequence Archive (GSA, https://ngdc.cncb.ac.cn/gsa) in NGDC, under accession number CRA004363 with BioProject ID PRJCA005451 [36]. In the *de novo* prediction, Augustus v.3.3.1 [89], GENSCAN [90] and GlimmerHMM [91] were used to analyze the masked genome and perform gene predictions. Finally, EVidenceModeler [92] was used to integrate all predictions and produce non-redundant and consensus gene sets, with 22,574 gene models in *R. mucronata* and 23,545 in *R. stylosa* annotated (Table S1).

Genome completeness (95.2% for *R. mucronta* and 95.9% for *R. stylosa*) was assessed by mapping the BUSCOs of the eudicotyledons_odb10 database (<https://github.com/c-omics/busco>, Table S1) back to the assemblies. We also mapped raw reads of the Illumina sequencing to the genome assemblies using the Burrows-Wheeler Aligner (BWA) [93,94]. 94.52% of *R. mucronta* reads and 92.53% of *R. stylosa* reads were successfully mapped to the corresponding assembly (Table S2).

***Collinearity analysis***

We performed collinearity analyses within and between *R. mucronata* and *R. stylosa* genomes. Alignment was performed on protein sequences using BLASTP (with a cutoff e-value of 10^−10^, identity ≥ 40%). We then used MCScanX [95] to identify syntenic (i.e. collinear) blocks, with blocks containing at least five paired homologous genes accepted. Genomic distribution of collinear blocks was visualized using the Circos (v0.65) software (Fig. 1E and Figs. S1-S2) [96]. 310 and 382 syntenic blocks were identified within the *R. mucronata* and *R. stylosa* genome, occupying up to 39.37% (8,887 of all 22,574 genes) and 40.54% (9,545 of all 23,545 genes) genes of each genome (Figs. S1-S2). Through collinearity analysis between *R. mucronata* and *R. stylosa*, we found 838 inter-specific collinear blocks with 18,118 *R. mucronata* (80.26% of 22,574 genes) and 18,553 *R. stylosa* genes (78.80% of 23,545 genes) involved (Fig. 1E).

The Ks values of pairs of intraspecific and interspecific collinear genes were calculated using KaKs_Calculator 2.0 (with the model YN-HKY) [97]. We also used the interspecific collinear genes to compute the *D_xy_* values of *R. mucronata* and *R. stylosa* with an in-house script, following the equation in the book of Gillespie,1998 [98]:

*D_xy_* = $\frac{1}{e} \sum_{i=1}^{k} {[p}_{i1}\left( 1-p_{i2} \right)+p_{i2}(1-p_{i1})]$

Where e means the effective length (number of all effective sites with mapped reads depth >=2) of the region or a window; k means the number of segregating sites (SNPs); $p_{1}$ and $p_{2}$ are derived allele frequency of population 1 and population 2, and $p_{\mathrm{ij}}$ is the observed frequency of SNP i in population j. The *D_xy_* value ranges from 0 to 1, a *D_xy_* of indicates no genetic difference between the two target populations while 1 indicates complete differentiation.

***Gene family analysis and divergence time estimation***

To estimate divergence times, we used the *de novo* genomes of *Carallia pectinifolia* [36], *Bruguiera gymnorrhiza* [32], *Rhizophora apiculata* [30], *R. mucronata*, and *R. stylosa*. We used OrthoFinder-2.2.7 [99] to identify gene families [94,100]. All proteins from these five species were merged to perform an all-to-all alignment using DIAMOND (with a cutoff e-value of 10^−30^) [101]. 20,971 gene families were identified among the five species, 6,014 containing only one gene in each species (i.e., single-copy orthologs). Genes from the five species that fell into shared single-copy orthologs were aligned using MUSCLE [102] and codon sequences were obtained using PAL2NAL.v14 [103]. We used JMODELTEST2 [104] to select an appropriate nucleotide-substitution model for reconstructing the phylogeny. To reconstruct the phylogenetic tree, we used RAXML [37] and IQTREE [38] with the best-fit model (GTR+G) and 1000 bootstrap replicates (Fig. S3).

Finally, the program MCMCTREE from the PAML4.8 package [105] was employed to estimate divergence times (Table S3 and Fig. S3), using "seq like (usedata = 1)", "JC69 (model = 0, alpha = 0)", "independent rates (clock = 2). "2000000 iterations (sampfreq = 10 and nsample = 200000)". The pollen fossils of *Rhizophora*, which were assigned an age of upper Eocene, were used to constrain the crown age of the clade consists of *Rhizophora* species and *Bruguiera gymnorhiza* to >38.0 Mya [106–108].

***Sampling and genome re-sequencing***

To make the samples of *R. mucronata* and *R. stylosa* more representative, we collected individuals both in allopatry and sympatry in the Indo-West Pacific region (Fig. 2 and Table S4). We re-sequenced 31 *R. mucronata* individuals from seven populations and 21 *R. stylosa* individuals from four populations (Fig. 2 and Tables S4-S6). To tell apart the two species by morphology, we observed the style length and shape in the bud and took photos (Fig. 3 and Tables S9). Fresh leaves were sampled from individual trees and dried with silica gel. Genomic DNA extraction was done following the CTAB method [70]. Short-read libraries were sequenced using the Illumina Hiseq 2000 platform with insert size of 350 bp and constructed following the TruSeq DNA Sample Preparation Guide. We obtained high quality sequence data for each individual genome, with coverage in the 12 to 22X range (Tables S5-S6).

***SNP calling and genetic diversity detection***

To identify variants, clean reads from all 52 individuals were mapped to the *de novo* *R. mucronata* genome using the Burrows-Wheeler Aligner (BWA) [93]. SAMtools [109] were used to import, sort, and pair bam files and remove duplications. To obtain high quality variants, single nucleotide polymorphisms (SNPs) were called and filtered using the Genome Analysis Toolkit (GATK) [110] and the SAMtools/bcftools [109] pipeline. Only consensus SNPs called by both pipelines were retained for downstream analyses. To remove low-quality variants, we eliminated all loci that had base quality (Q) or mapping quality (q) smaller than 20. We additionally performed the following stringent filtering: 1) at least two reads had to support the minor allele to call a heterozygote; 2) only homozygous SNPs with read depth >=2 were retained. After filtering, we selected these high-quality sites for further analyses, with multi-allelic (>=3) sites, insertions, and deletions excluded. To estimate genetic diversity in each population, we calculated θ_w_ (Watterson’s θ_w_) and θ_π_ (Nei and Li’s θ_π_) within each population following the equations of [111,112], with an in-house script (Table S4). To estimate genomic divergence between *R. mucronata* and *R. stylosa* populations, we calculated the genetic differentiation coefficient (*F_ST_*) following the equation of [111,113], with an in-house script (Fig. 2D).

***Genomic scan for introgressed and non-introgressable blocks***

We used four predefined taxa: m1 (*R. mucronata* population in Daintree River with 5 individuals), s1 (*R. stylosa* population in Daintree River with 5 individuals), M_allo_ (allopatric *R. mucronata* populations m2-m7 with 26 individuals), and S_allo_ (allopatric *R. stylosa* populations s2-s4 with 16 individuals). To get a more informative data set, we filtered sites with large number of missing genotypes in each taxon (> 8 in M_allo_, > 6 in S_allo_, > 2 in m1 or > 2 in s1) or low divergence (*F_ST_* <= 0.8) between M_allo_ and S_allo_. We retained 305,418 SNPs (*F_ST_* > 0.8) which we call divergent sites or d-sites between M_allo_ and S_allo_ (Fig. 2C). 212,626 of the d-sites are fixed (*F_ST_* = 1.0 and *D_xy_* = 1.0) between M_allo_ and S_allo_ (Fig. 2C). There are four possible states of each d-site: homozygous *R. mucronata* variant (MM), homozygous *R. stylosa* variant (SS), heterozygote (MS), or missing data.

We developed a novel pipeline to identify introgressed sites (i-sites) and non-introgressable sites (j-sites) among all the d-sites across m1 and s1 genomes. Our method followed the principle used in the popular tool ABBA-BABA (or *D*) statistics, which was designed to detect the excess of shared derived alleles in the sympatric (DR) population in *R. mucronata* (m1) or *R. stylosa* (s1) in relative to the allopatric *R. mucronata* populations m2-m7 (M_allo_) and allopatric *R. stylosa* populations s2-s4 (S_allo_). To be conservative, we constrained the detection only to the highly divergent sites (d-sites with *F_ST_* > 0.8) between Mallo and Sallo. At each of such d-sites, we identified m1 alleles derived from *R. stylosa* and s1 alleles derived from *R. mucronata*. As we have five diploid individuals (or 10 haploid genomes) from the m1 and s1 populations, we defined some terms as follows:

**Introgressed allele** (**i-allele)**: *R. stylosa* variant in m1 populations or *R. mucronata* variant in s1 populations.

**i-site:** an i-site in m1 or in s1 genomes is defined as >= 8 occurrences of i-allele out of the 10 genomes (Fig. 4B and Fig. S10).

**j-site:** a d-site with <=1 occurrences of i-allele in both m1 and s1 populations (Fig. 4B and Fig. S10).

**i-block:** A genomic block in one species is considered introgressed from the other species if one or more i-sites continuously (without disruption by other d-sites) are present (Fig. 4C and Figs. S11 and S13). The length of an i-block is determined by the midpoint between the flanking (d-sites, i-sites) intervals (as shown in Fig. 4C).

**j-block:** a genomic block with one or more j-sites continuously. We define the boundaries the same as for i-blocks.

***Simulations of genomic sequences under hybridization, selection, and recombination***

To probe the influences of hybridization, selection, and recombination on genomic sequences, we carried out computer simulations based on the Recurrent Selection and Backcross (RSB) model [51]. We set high and low levels for each parameter. Population size was set at 1000. The length of simulated sequences was 100 Kb (for convenience, 1 Kb is the basic unit that cannot be separated by recombination). The original allele in the sequence and an i-allele from the other species were differentially labeled. Hence, at the beginning of the simulations, the sequences of all individuals were in original allelic states (100 x). After several generations of hybridization, selection, and recombination, the sequences become shuffled (Figs. S13-S14).

We first set a low hybridization rate (introgression or migration rate, m = 0.001 per generation) and recombination (10E-6 per generation between adjacent base pairs). For every generation, 999 individuals were picked from the original population and one from the other population (or species). The recombination probability (r) for a 100 Kb sequence was about 0.1. Since population size is 1000, there will be an average of 100 individuals with recombination in each generation. Two loci under negative selection (#51 and #71) were defined in the simulated sequences. If one or both loci harbor an i-allele, the relative fitness of this sequence is 0.95 (Fig. S14A) or 0.99 (Fig. S14B). We also examined a high introgression rate regime (10/1000). In this case, four loci (#41, #51, #71 and #76) were negatively selected (relative fitness = 0.95 for an i-allele) (Fig. S14C). The scenarios in Fig. S14A show that a lower recombination rate (r = 0.1) increases the size of non-introgressed DNA segments, because the neutral genes near positions 51 and 71 were selected against along with the speciation loci. Figure S14B- S14C shows that a reduced selection intensity (s = -0.01) or a 10-fold higher introgression rate give rise to extensive introgressions. Interestingly, partial introgressions were detected even at positions 51 and 71, where selection acts against the invading alleles.

Finally, we simulated genomic sequences under a high recombination rate (10E-5, r = 1.0 for a 100 Kb simulated sequence per generation) and a low introgression rate (1/1000 per generation). Two loci (#51 and #71) were negatively selected (relative fitness = 0.95 for an i-allele) (Fig. S13D-S13E and Fig. S14D-S14F). The simulations suggest that, given the right parameter values, the pattern of introgression would follow exactly the prediction based solely on selection, whereby only the alleles of the speciation loci cannot be introgressed (Fig. S14D-S14F). The rest of the genome, even right next to the speciation loci, is freely shared between species (Fig. S14D-S14F).

**Supplementary Tables:**

**Table S1 *De novo* sequencing and assembly of *R. mucronata* and *R. stylosa* genomes**

|  | ***R. mucronata*** | ***R. stylosa*** |
| --- | --- | --- |
| Total assembly size (Mb) | 237.85 | 219.67 |
| Total number of contigs | 18,591 | 22,741 |
| Total size of contigs >=1000 bp (Mb) | 235.87 | 246.80 |
| The longest contig (Mb) | 3.14 | 3.85 |
| No. contigs (length >=1 Mb) | 37 | 16 |
| Contig N50 length (Kb) | 385.92 | 153.43 |
| Total number of scaffolds | 14,496 | 317 |
| The longest scaffold (Mb) | 16.96 | 19.12 |
| The shortest scaffold (bp) | 1,000 | 20,16 |
| No. scaffolds (length >= 5 Mb) | 18 | 18 |
| Total length of the top 18 longest scaffolds (Mb) | 200.70 | 218.34 |
| Proportion of the assembly genome **^a^** | 84.38% | 99.39% |
| Scaffold N50 length (Mb) | 12.03 | 12.64 |
| Scaffold N50 count | 9 | 8 |
| Total N (Mb) | 14.72 | 23.54 |
| GC content (%) | 35.46 | 35.78 |
| No. annotation genes | 22,574 | 23,545 |
| Repeat content (%) | 28.39 | 24.61 |
| % of BUSCOs mapped to the genome assembly**^b^** | 95.2% | 95.9% |
| % of BUSCOs mapped to the gene models **^b^** | 89.5% | 91.4% |

**^a^** Proportion occupied by the top 18 longest scaffolds of the assembly genome.

**^b^** Genomic/protein completeness was assessed with the lineage database eudicotyledons_odb10, using BUSCO (https://github.com/c-omics/busco). There are 2,326 BUSCOs in the dataset.

**Table S2 *De novo* sequencing information and genetic statistics of the *R. mucronata* and**

***R. stylosa* genomes**

|  | ***R. mucronata*** | ***R. stylosa*** |
| --- | --- | --- |
| Sampling location (Longitude, latitude) | Dongzhai Harbor, Hainan, China (110°35'5.79'' E, 19°56'39.67'' N) | Dongzhai Harbor,Hainan, China (110°35'5.79'' E, 19°56'39.67'' N) |
| Sample ID | RM | RS |
| Read length (bp) | 100 | 100 |
| Raw read pairs | 169,210,696 | 180,644,954 |
| Retained clean reads pairs | 141,318,480 | 153,186,318 |
| Mapped reads | 267,136,374 | 284,531,021 |
| Reads mapping rate (%) | 94.52 | 92.53 |
| Assembly genome size (bp) | 237,845,386 | 219,666,082 |
| Effective sites **^a^** | 196,713,446 | 191,597,001 |
| Coverage rate **^b^** | 0.83 | 0.87 |
| Mean depth **^c^** | 142 | 164 |
| No. of heterozygotes | 94,170 | 82,823 |
| Ht **^d^** (per Kb) | 0.48 | 0.43 |
| No. private heterozygotes | 37,923 | 48,766 |
| No. shared heterozygotes | 46,218 | |
| ***D_xy_* (RM vs. RS)** | **0.0031** | |

**^a^** Effective sites: the sites that have at least two reads mapped (depth>=2) for each de novo sequencing individual.

**^b^** Coverage rate: the proportion occupied by effective sites of the assembly genome size.

**^c^** Mean depth is (raw reads*reads length)/assembly genome size.

**^d^** Mean genome-wide heterozygosity (Ht): the proportion of all effective sites occupied by heterozygotes.

**Table S3 Divergence time and credible interval of each node of the Rhizophoreae group**

**using *MCMCTREE***

| **Node** | **Divergence time (Mya)** | **95%HPD ^a^ (Mya)** |
| --- | --- | --- |
| 1 | 2.70 | [2.05, 3.44] |
| 2 | 8.51 | [6.53, 10.52] |
| 3 | 42.76 | [37.77, 47.94] |
| 4 | 67.59 | [51.64, 82.42] |

**^a^** 95%HPD: 95% highest posterior density credible interval of each node is shown. Nodes are marked in Supplementary Fig. S3b. Mya: million years ago.

**Table S4 Sampling, re-sequencing information, and genetic diversity statistics for**

***R. mucronata* and *R. stylosa* populations**

| **Sampling location** | **Longitude, latitude** | **Pop ID** | **Sample size** | **Effective sites ^a^** | **Coverage rate ^b^** | **SNPs (E+05)** | **θ_w_ (Kb)** | **θ_π_ (Kb)** |
| --- | --- | --- | --- | --- | --- | --- | --- | --- |
| ***R. mucronata*** |  |  |  |  |  |  |  |  |
| Daintree River, Australia | 145°26'16.28'' E,16°17'12.44'' S | m1 | 5 | 1.98E+08 | 0.83 | 7.80 | 1.39 | 1.37 |
| Saint John's Island, Singapore | 103°50'30.19'' E, 1°13'6.60'' N | m2 | 5 | 1.92E+08 | 0.81 | 2.81 | 0.52 | 0.65 |
| Chai-Ya, Thailand | 99°15'32.35'' E, 9°21'23.07'' N | m3 | 4 | 1.91E+08 | 0.80 | 2.02 | 0.41 | 0.49 |
| Ranong, Thailand | 98°37'26.01'' E, 9°57'36.26'' N | m4 | 4 | 1.95E+08 | 0.82 | 5.12 | 1.01 | 1.17 |
| Tanjung Piai, Malaysia | 103°21'1.86'' E, 1°24'8.11'' N | m5 | 5 | 1.95E+08 | 0.82 | 3.53 | 0.64 | 0.74 |
| Mauritius | 57°41'18.33'' E, 20°20'26.37'' S | m6 | 3 | 1.90E+08 | 0.80 | 0.86 | 0.20 | 0.24 |
| Kenya | 39°36'1.82'' E, 4°24'24.15'' S | m7 | 5 | 1.98E+08 | 0.83 | 1.90 | 0.34 | 0.42 |
| ***R. stylosa*** |  |  |  |  |  |  |  |  |
| Daintree River, Australia | 145°26'16.28'' E, 16°17'12.44'' S | s1 | 5 | 1.95E+08 | 0.82 | 9.05 | 1.64 | 2.09 |
| Saint John's Island, Singapore | 103°50'30.19'' E, 1°13'6.60'' N | s2 | 5 | 1.90E+08 | 0.80 | 1.28 | 0.24 | 0.30 |
| Darwin, Australia | 130°54'22.64'' E, 12°25'5.75'' S | s3 | 6 | 1.89E+08 | 0.80 | 5.99 | 1.05 | 1.23 |
| Hainan, China | 110°35'5.79'' E, 19°56'39.67'' N | s4 | 5 | 1.93E+08 | 0.81 | 1.19 | 0.22 | 0.28 |

**^a^** Effective sites: the sites that have at least one individual mapped (depth>=2) for each population.

**^b^** Coverage rate: the proportion occupied by effective sites of the reference genome size. The reference (*R. mucronata*) genome size is 237,845,386 bp.

**Table S5 Re-sequencing characteristics and heterozygosity of each *R. mucronata* genome**

| **Population**  **ID** | **Ind. ID** | **Read length (bp)** | **Raw read pairs (E+07)** | **Clean read pairs (E+07)** | **Mapped reads (E+07)** | **Reads mapping rate (%)** | **Effective sites ^a^ (E+08)** | **Coverage rate ^b^** | **Mean Depth ^c^** | **Ht ^d^ (per Kb)** |
| --- | --- | --- | --- | --- | --- | --- | --- | --- | --- | --- |
| **m1**  (Daintree River, Australia) | m1-1 | 125 | 2.03 | 2.03 | 3.58 | 88.33 | 1.93 | 0.81 | 19X | 2.00 |
|  | m1-15 | 125 | 1.37 | 1.37 | 2.51 | 91.32 | 1.89 | 0.79 | 13X | 0.47 |
|  | m1-16 | 125 | 1.36 | 1.36 | 2.40 | 88.09 | 1.90 | 0.80 | 13X | 2.09 |
|  | m1-2 | 100 | 2.18 | 1.99 | 3.68 | 92.45 | 1.78 | 0.75 | 15X | 0.50 |
|  | m1-3 | 100 | 2.51 | 2.28 | 4.21 | 92.28 | 1.81 | 0.76 | 18X | 0.38 |
| **m2**  (Saint John's Island, Singapore) | m2-1 | 150 | 1.49 | 1.43 | 2.56 | 89.71 | 1.82 | 0.76 | 16X | 0.80 |
|  | m2-2 | 150 | 2.09 | 1.98 | 3.53 | 89.02 | 1.85 | 0.78 | 22X | 0.42 |
|  | m2-3 | 150 | 1.62 | 1.58 | 2.96 | 93.90 | 1.81 | 0.76 | 19X | 0.60 |
|  | m2-4 | 150 | 1.44 | 1.40 | 2.64 | 94.42 | 1.83 | 0.77 | 17X | 0.65 |
|  | m2-5 | 150 | 1.78 | 1.73 | 3.25 | 93.79 | 1.85 | 0.78 | 20X | 0.66 |
| **m3**  (Chai-Ya, Thailand) | m3-12 | 125 | 1.48 | 1.48 | 2.75 | 92.55 | 1.84 | 0.77 | 14X | 0.46 |
|  | m3-14 | 125 | 1.62 | 1.62 | 3.03 | 93.75 | 1.83 | 0.77 | 16X | 0.50 |
|  | m3-5 | 125 | 1.51 | 1.51 | 2.85 | 94.07 | 1.86 | 0.78 | 15X | 0.46 |
|  | m3-7 | 125 | 1.42 | 1.42 | 2.65 | 93.38 | 1.84 | 0.77 | 14X | 0.50 |
| **m4**  (Ranong, Thailand) | m4-1 | 125 | 1.70 | 1.70 | 3.16 | 92.91 | 1.85 | 0.78 | 17X | 0.45 |
|  | m4-2 | 125 | 1.46 | 1.46 | 2.71 | 92.79 | 1.85 | 0.78 | 14X | 0.63 |
|  | m4-3 | 125 | 1.65 | 1.65 | 3.08 | 93.13 | 1.83 | 0.77 | 16X | 0.62 |
|  | m4-4 | 125 | 1.49 | 1.49 | 2.76 | 92.86 | 1.89 | 0.79 | 15X | 0.67 |
| **m5**  (Tanjung Piai, Malaysia) | m5-12 | 125 | 1.40 | 1.34 | 2.49 | 93.02 | 1.90 | 0.80 | 13X | 0.85 |
|  | m5-14 | 125 | 1.90 | 1.90 | 3.33 | 87.41 | 1.82 | 0.76 | 17X | 0.44 |
|  | m5-21 | 125 | 2.01 | 2.01 | 3.60 | 89.43 | 1.79 | 0.75 | 19X | 0.65 |
|  | m5-32 | 125 | 1.34 | 1.28 | 2.38 | 92.90 | 1.79 | 0.75 | 13X | 0.84 |
|  | m5-9 | 125 | 1.27 | 1.19 | 2.20 | 92.88 | 1.78 | 0.75 | 12X | 0.52 |
| **m6**  (Mauritius) | m6-1 | 125 | 1.35 | 1.35 | 2.47 | 91.44 | 1.77 | 0.74 | 13X | 0.38 |
|  | m6-2 | 125 | 1.34 | 1.34 | 2.51 | 93.88 | 1.83 | 0.77 | 13X | 0.34 |
|  | m6-3 | 125 | 1.32 | 1.32 | 2.42 | 91.90 | 1.82 | 0.76 | 13X | 0.35 |
| **m7**  (Kenya) | m7-10 | 125 | 1.55 | 1.55 | 2.79 | 89.77 | 1.84 | 0.78 | 15X | 0.51 |
|  | m7-11 | 125 | 1.48 | 1.48 | 2.59 | 87.58 | 1.85 | 0.78 | 14X | 0.52 |
|  | m7-2 | 125 | 1.77 | 1.77 | 3.11 | 87.57 | 1.83 | 0.77 | 16X | 0.41 |
|  | m7-7 | 125 | 2.11 | 2.11 | 3.75 | 88.80 | 1.90 | 0.80 | 20X | 0.52 |
|  | m7-9 | 125 | 1.92 | 1.92 | 3.38 | 87.74 | 1.83 | 0.77 | 18X | 0.53 |

**^a^** Effective sites: all sites that have at least two reads mapped (depth>=2) in each site.

**^b^** Coverage rate: the proportion occupied by effective sites of the reference genome size. The reference (*R. mucronata*) genome size equals to 237,845,386 bp.

**^c^** Mean depth is (mapped reads*reads length)/reference genome size.

**^d^** Mean genome-wide heterozygosity (Ht): the proportion of all effective sites occupied by heterozygotes.

**Table S6 Re-sequencing characteristics and heterozygosity of each *R. stylosa* genome**

| **Population**  **ID** | **Ind. ID** | **Read length (bp)** | **Raw read pairs (E+07)** | **Clean read pairs (E+07)** | **Mapped reads (E+07)** | **Reads mapping rate (%)** | **Effective sites ^a^ (E+08)** | **Coverage rate ^b^** | **Mean Depth ^c^** | **Ht ^d^ (per Kb)** |
| --- | --- | --- | --- | --- | --- | --- | --- | --- | --- | --- |
| **s1**  (Daintree River, Australia) | **s1-10** | 100 | 1.83 | 1.68 | 3.11 | 92.80 | 1.85 | 0.78 | 13X | 3.32 |
|  | **s1-5** | 100 | 1.72 | 1.58 | 2.91 | 92.24 | 1.89 | 0.80 | 12X | 1.58 |
|  | **s1-6** | 100 | 1.95 | 1.79 | 3.27 | 91.31 | 1.85 | 0.78 | 14X | 2.03 |
|  | **s1-7** | 100 | 2.01 | 1.85 | 3.42 | 92.74 | 1.78 | 0.75 | 14X | 3.43 |
|  | **s1-9** | 100 | 1.81 | 1.66 | 3.07 | 92.20 | 1.82 | 0.77 | 13X | 2.04 |
| **s2**  (Saint John's Island, Singapore) | **s2-1** | 150 | 1.58 | 1.54 | 2.88 | 93.93 | 1.77 | 0.74 | 18X | 0.40 |
|  | **s2-3** | 150 | 1.72 | 1.68 | 3.17 | 94.57 | 1.80 | 0.76 | 20X | 0.40 |
|  | **s2-4** | 150 | 1.53 | 1.48 | 2.76 | 93.18 | 1.80 | 0.76 | 17X | 0.39 |
|  | **s2-5** | 150 | 1.41 | 1.38 | 2.60 | 94.05 | 1.81 | 0.76 | 16X | 0.38 |
|  | **s2-6** | 150 | 1.43 | 1.39 | 2.64 | 94.61 | 1.81 | 0.76 | 17X | 0.40 |
| **s3**  (Darwin, Australia) | **s3-1** | 100 | 2.24 | 2.07 | 3.76 | 91.04 | 1.78 | 0.75 | 16X | 1.27 |
|  | **s3-2** | 100 | 2.19 | 2.01 | 3.59 | 89.47 | 1.80 | 0.76 | 15X | 1.23 |
|  | **s3-3** | 100 | 1.94 | 1.80 | 3.24 | 90.07 | 1.81 | 0.76 | 14X | 0.78 |
|  | **s3-4** | 100 | 2.16 | 1.97 | 3.46 | 87.64 | 1.83 | 0.77 | 15X | 1.18 |
|  | **s3-5** | 100 | 2.26 | 2.07 | 3.74 | 90.38 | 1.79 | 0.75 | 16X | 1.26 |
|  | **s3-6** | 100 | 2.69 | 2.46 | 4.46 | 90.68 | 1.81 | 0.76 | 19X | 1.31 |
| **s4**  (Hainan, China) | **s4-10** | 100 | 1.99 | 1.82 | 3.33 | 91.42 | 1.80 | 0.76 | 14X | 0.40 |
|  | **s4-13** | 100 | 2.00 | 1.84 | 3.34 | 90.85 | 1.79 | 0.75 | 14X | 0.37 |
|  | **s4-3** | 100 | 1.85 | 1.69 | 3.12 | 91.98 | 1.79 | 0.75 | 13X | 0.37 |
|  | **s4-4** | 100 | 1.79 | 1.65 | 3.04 | 92.24 | 1.76 | 0.74 | 13X | 0.39 |
|  | **s4-7** | 100 | 1.91 | 1.75 | 3.22 | 91.79 | 1.86 | 0.78 | 14X | 0.35 |

**^a^**Effective sites: all sites that have at least two reads mapped (depth>=2) in each site.

**^b^** Coverage rate: the proportion occupied by effective sites of the reference genome size. The reference (*R. mucronata*) genome size equals to 237,845,386 bp.

**^c^** Mean depth is (mapped reads*reads length)/reference genome size.

**^d^** Mean genome-wide heterozygosity (Ht): the proportion of all effective sites occupied by heterozygotes.

**Table S7 Genome-wide genetic divergence between and within *R. mucronata* and**

***R. stylosa* populations**

| **Description** | **Relationship** | **Mean genome-wide**  ***D_xy_* (per Kb)** | **Mean genome-wide**  ***F_ST_*** |
| --- | --- | --- | --- |
| Sympatric divergence | **m1 vs. s1** | **2.40** | **0.21** |
| Genetic divergence  within *R. mucronata* | m1 vs. M_allo_ **^a^** | 3.41 | 0.26 |
| Genetic divergence  within *R. stylosa* | s1 vs. S_allo_ **^b^** | 2.90 | 0.25 |
| Genetic divergence between  interspecific populations | m1 vs. S_allo_ | 3.94 | 0.43 |
|  | s1 vs. M_allo_ | 3.90 | 0.27 |
|  | **M_allo_ vs. S_allo_** | **4.37** | **0.49** |
| Genetic divergence between  *R. mucronata* and *R. stylosa* | **M_all_ vs. S_all_ ^c^** | **4.14** | **0.34** |

**^a^** "M_allo_": allopatric *R. mucronata* populations m2-m7.

**^b^** "S_allo_": allopatric *R. stylosa* populations s2-s4.

**^c^** "M_all_" represents all *R. mucronata* populations m1-m7, while "S_all_" represents all *R. stylosa* populations s1-s4.

**Table S8 *R. mucronata* and *R. stylosa* polymorphism statistics**

| **Removed**  **populations ^a^** | **Fixed**  **difference** | **Shared**  **polymorphisms** | **Private**  **polymorphisms**  **in *R. stylosa*** | **Private**  **polymorphisms**  **in *R. mucronata*** | **Total**  **SNPs** |
| --- | --- | --- | --- | --- | --- |
| none | 325 | 900,474 | 322,965 | 517,259 | 1,741,023 |
| **m1, s1** | **215,350** | **259,759** | **492,260** | **590,503** | **1,741,023** |
| m2, s2 | 343 | 885,312 | 314,701 | 518,424 | 1,741,023 |
| m2, s3 | 418 | 845,759 | 207,212 | 557,977 | 1,741,023 |
| m2, s4 | 341 | 886,514 | 311,177 | 517,222 | 1,741,023 |
| m3, s2 | 342 | 887,920 | 312,093 | 523,907 | 1,741,023 |
| m3, s3 | 417 | 848,259 | 204,712 | 563,568 | 1,741,023 |
| m3, s4 | 340 | 889,211 | 308,480 | 522,616 | 1,741,023 |
| m4, s2 | 370 | 881,242 | 318,771 | 459,051 | 1,741,023 |
| m4, s3 | 445 | 843,004 | 209,967 | 497,289 | 1,741,023 |
| m4, s4 | 366 | 882,372 | 315,319 | 457,921 | 1,741,023 |
| m5, s2 | 351 | 885,605 | 314,408 | 513,202 | 1,741,023 |
| m5, s3 | 422 | 846,239 | 206,732 | 552,568 | 1,741,023 |
| m5, s4 | 346 | 886,887 | 310,804 | 511,920 | 1,741,023 |
| m6, s2 | 346 | 886,636 | 313,377 | 518,285 | 1,741,023 |
| m6, s3 | 420 | 847,372 | 205,599 | 557,549 | 1,741,023 |
| m6, s4 | 343 | 887,946 | 309,745 | 516,975 | 1,741,023 |
| m7, s2 | 356 | 881,564 | 318,449 | 504,829 | 1,741,023 |
| m7, s3 | 432 | 844,254 | 208,717 | 542,139 | 1,741,023 |
| m7, s4 | 354 | 882,873 | 314,818 | 503,520 | 1,741,023 |

**^a^** Removed populations: we removed two populations from all samples each time and then calculate the polymorphisms in the rest *R. mucronata* and *R. stylosa* populations.

"none" means we kept all samples.

**Table S9 Additional diagnostic morphological features to identify *R. mucronata* and *R. stylosa***

| **Feature** | ***R. mucronata*** | ***R. stylosa*** |
| --- | --- | --- |
| **bracts and bracteoles** | minute bracts and bracteoles | distinct bracts and bracteoles |
| **inflorescences** | 1-2 flowered inflorescences | 4-16 flowered inflorescences |
| **flower buds** | irregular obovoid closed flower buds | regular ovoid-elliptic closed flower buds |
| **propagules** | long propagules reaching ~80 cm | ~60 cm |

**Table S10 Patterson’s *D* statistic and improved *f_d_* statistic, showing evidence of gene flow between**

***R. mucronata* (m1) and *R. stylosa* (s1) in sympatry in Daintree River, Australia**

| **Model code ^a^** | **Pop1 ^b^** | **Pop2 ^b^** | **Pop3 ^b^** | **Outgr-oup ^b^** | ***D* ± std err ^c^** | **Z-score** | **P-value** | ***f_d_* ± std err ^d^** |
| --- | --- | --- | --- | --- | --- | --- | --- | --- |
| **1** | m7 | m6 | s1 | ra | -0.0272 ± 0.0151 | -0.0890 | 0.929 | -0.00517 ± 4.54E-05 |
| **2** | m7 | m6 | s2 | ra | 0.0138 ± 0.0216 | 0.0316 | 0.975 | 0.00284 ± 7.78 E-05 |
| **3** | m7 | m6 | s3 | ra | -0.0136 ± 0.0206 | -0.0327 | 0.974 | -0.00226 ± 7.70E-05 |
| **4** | m7 | m6 | s4 | ra | -0.00350 ± 0.0216 | -0.00798 | 0.994 | 0.00161 ± 7.64E-05 |
| **5** | m3 | m2 | s2 | ra | -0.00127 ± 0.0135 | -0.00466 | 0.996 | -0.00200 ± 2.57E-05 |
| **6** | m4 | m2 | s2 | ra | 0.113 ± 0.0140 | 0.399 | 0.690 | 0.0108 ± 4.85E-05 |
| **7** | m5 | m2 | s2 | ra | 0.00626 ± 0.0120 | 0.0257 | 0.979 | -0.000231 ± 5.06E-06 |
| **8** | m6 | m2 | s2 | ra | -0.169 ± 0.0209 | -0.401 | 0.688 | -0.0333 ± 1.61E-04 |
| **9** | m7 | m2 | s2 | ra | -0.174 ± 0.0209 | -0.412 | 0.681 | -0.0309 ± 2.16E-04 |
| **10** | s3 | s2 | m2 | ra | 0.128 ± 0.0180 | 0.351 | 0.726 | 0.0409 ± 1.71E-04 |
| **11** | s4 | s2 | m2 | ra | 0.00597 ± 0.0209 | 0.0141 | 0.989 | -0.0137 ± 1.10E-04 |
| **12** | m2 | m1 | s1 | ra | 0.640 ± 0.00861 | 3.67*** | **2.42E-04** | 0.445 ± 7.23E-05 |
| **13** | m3 | m1 | s1 | ra | 0.637 ± 0.00894 | 3.52*** | **4.32E-04** | 0.442 ± 7.43E-05 |
| **14** | m4 | m1 | s1 | ra | 0.646 ± 0.0101 | 3.17*** | **1.51E-03** | 0.447 ±7.77E-05 |
| **15** | m5 | m1 | s1 | ra | 0.642 ± 0.00832 | 3.81*** | **1.38E-04** | 0.445 ± 7.36E-05 |
| **16** | m6 | m1 | s1 | ra | 0.565 ± 0.0103 | 2.70*** | **6.89E-03** | 0.422 ± 8.39E-05 |
| **17** | m7 | m1 | s1 | ra | 0.561 ± 0.0102 | 2.71*** | **6.65E-03** | 0.420 ± 8.87E-05 |
| **18** | s2 | s1 | m1 | ra | 0.607 ± 0.00949 | 3.16*** | **1.59E-03** | 0.548 ± 1.14E-04 |
| **19** | s3 | s1 | m1 | ra | 0.600 ± 0.00912 | 3.24*** | **1.18E-03** | 0.553 ± 8.49E-05 |
| **20** | s4 | s1 | m1 | ra | 0.605 ± 0.00951 | 3.14*** | **1.67E-03** | 0.544 ± 9.83E-05 |

**^a^**: code of *D* statistic models. Models 1-11 exclude sympatric populations m1 and s1; models 12-20 contain sympatric populations m1 and s1.

**^b^**: Pop1, Pop2, Pop3 and Outgroup respectively refer to the three ingroups and the outgroup (ra: *R. apiculata*) following the genealogical relationship (((Pop1, Pop2), Pop3), Outgroup).

**^c^**: *D* statistic, given as a ratio *D* ± standard error.

***: the genome-wide average *D*-statistic value *D* is significantly derived from 0 with P < 0.01, indicating the existence of gene flow between m1 and s1 populations.

**^d^**: *f_d_* statistic, given as an admixed proportion *f_d_* ± standard error.

**Table S11 Introgressed site (i-site) distribution across introgressed blocks (or i-blocks) in m1 and s1 genomes**

| **The i-sites range**  **of i-blocks** | **>=2 occurrences**  **of i-allele** | | **>=4 occurrences**  **of i-allele** | | **>=6 occurrences**  **of i-allele** | | **>=8 occurrences**  **of i-allele** | | **=10 occurrences**  **of i-allele** | |
| --- | --- | --- | --- | --- | --- | --- | --- | --- | --- | --- |
|  | **m1** | **s1** | **m1** | **s1** | **m1** | **s1** | **m1** | **s1** | **m1** | **s1** |
| 1 (singleton block) | 17,961 | 8,912 | 17,195 | 8,023 | 17,246 | 7,226 | 17,278 | 5,411 | 17,091 | 3,333 |
| 2 | 4,937 | 3,004 | 4,878 | 2,790 | 4,957 | 2,341 | 4,959 | 1,572 | 4,858 | 917 |
| 3 | 1,944 | 1,563 | 1,954 | 1,478 | 2,001 | 1,167 | 2,003 | 740 | 1,936 | 401 |
| 4 | 945 | 973 | 971 | 922 | 993 | 709 | 985 | 416 | 966 | 241 |
| 5 | 527 | 611 | 535 | 604 | 540 | 451 | 549 | 261 | 544 | 159 |
| 5-10 | 861 | 1,286 | 878 | 1,323 | 915 | 997 | 907 | 529 | 883 | 292 |
| 10-15 | 241 | 412 | 247 | 399 | 256 | 332 | 260 | 160 | 259 | 97 |
| 15-20 | 109 | 148 | 110 | 140 | 114 | 144 | 113 | 65 | 121 | 28 |
| 20-30 | 93 | 118 | 95 | 135 | 96 | 128 | 93 | 74 | 89 | 44 |
| 30-40 | 33 | 73 | 34 | 63 | 35 | 62 | 36 | 28 | 33 | 21 |
| 40-50 | 20 | 21 | 20 | 21 | 20 | 25 | 18 | 18 | 20 | 10 |
| 50-60 | 8 | 6 | 9 | 7 | 9 | 6 | 11 | 3 | 12 | 4 |
| 60-70 | 6 | 3 | 6 | 5 | 6 | 3 | 8 | 2 | 3 | 3 |
| 70-80 | 4 | 2 | 4 | 2 | 4 | 3 | 4 | 1 | 5 | 0 |
| 80-90 | 2 | 2 | 2 | 1 | 2 | 1 | 2 | 1 | 3 | 0 |
| 90-100 | 4 | 4 | 5 | 4 | 5 | 4 | 4 | 3 | 3 | 2 |
| >100 | 8 | 2 | 10 | 2 | 11 | 3 | 11 | 1 | 6 | 0 |
| Total blocks | 27,703 | 17,140 | 26,953 | 15,919 | 27,210 | 13,602 | 27,241 | 9,285 | 26,832 | 5,552 |
| Blocks (>=2 i-sites) | 9,742 | 8,228 | 9,758 | 7,896 | 9,964 | 6,376 | 9,963 | 3,874 | 9,741 | 2,219 |

**Table S12 Length distribution of introgressed blocks (i-blocks) in m1 and s1 genomes**

| **Length range of i-blocks** | **>=2 occurrences**  **of i-allele** | | **>=4 occurrences**  **of i-allele** | | **>=6 occurrences**  **of i-allele** | | **>=8 occurrences**  **of i-allele** | | **=10 occurrences**  **of i-allele** | |
| --- | --- | --- | --- | --- | --- | --- | --- | --- | --- | --- |
|  | **m1** | **s1** | **m1** | **s1** | **m1** |  | **m1** | **s1** | **m1** | **s1** |
| 1-10bp | 217 | 96 | 219 | 80 | 221 | 62 | 219 | 43 | 214 | 28 |
| 10-100bp | 5,144 | 2,337 | 4,998 | 2,051 | 5,104 | 1,817 | 5,113 | 1,298 | 5,073 | 705 |
| 100bp-1Kb | 15,690 | 9,096 | 15,149 | 8,268 | 15,276 | 7,021 | 15,309 | 4,649 | 15,180 | 2,754 |
| 1Kb-5Kb | 5,073 | 4,133 | 4,990 | 4,062 | 4,991 | 3,375 | 4,993 | 2,325 | 4,843 | 1,438 |
| 5Kb-10Kb | 787 | 839 | 791 | 800 | 801 | 726 | 797 | 521 | 786 | 337 |
| 10Kb-20Kb | 466 | 394 | 472 | 403 | 476 | 384 | 468 | 274 | 439 | 183 |
| 20Kb-30Kb | 155 | 97 | 158 | 110 | 161 | 88 | 163 | 75 | 143 | 44 |
| 30Kb-50Kb | 96 | 87 | 98 | 87 | 99 | 74 | 97 | 56 | 82 | 38 |
| 50Kb-100Kb | 55 | 43 | 57 | 41 | 59 | 41 | 57 | 35 | 50 | 20 |
| **>100Kb** | **20** | **18** | **21** | **17** | **22** | **14** | **25** | **9** | **22** | **5** |
| Total blocks | 27,703 | 17,140 | 26,953 | 15,919 | 27,210 | 13,602 | 27,241 | 9,285 | 26,832 | 5,552 |

**Table S13 Detailed information on introgressed blocks (i-blocks) in m1 and s1 genomes**

| **Description** | | **>=2 occurrences of**  **i-allele** | | **>=4 occurrences of**  **i-allele** | | **>=6 occurrences of**  **i-allele** | | **>=8 occurrences of**  **i-allele** | | **=10 occurrences of**  **i-allele** | |
| --- | --- | --- | --- | --- | --- | --- | --- | --- | --- | --- | --- |
|  |  | **m1 pop** | **s1 pop** | **m1 pop** | **s1 pop** | **m1 pop** |  | **m1 pop** | **s1 pop** | **m1 pop** | **s1 pop** |
| **The i-blocks contain >=1 intro sites** | No. of i-blocks | 27,703 | 17,140 | 26,953 | 15,919 | 27,210 | 13,602 | 27,241 | 9,285 | 26,832 | 5,552 |
|  | No. of scaffolds with i-blocks | 18 | 18 | 18 | 18 | 18 | 18 | 18 | 18 | 18 | 18 |
|  | Total length of i-blocks (Mb) | 45.33 | 39.61 | 45.50 | 38.80 | 46.07 | 33.61 | 46.33 | 24.21 | 43.14 | 15.57 |
|  | % of the genome | 22.58 | 19.74 | 22.67 | 19.33 | 22.96 | 16.75 | 23.09 | 12.06 | 21.49 | 7.76 |
| **The i-blocks contain >=2 intro sites** | No. of i-blocks | 9,742 | 8,228 | 9,758 | 7,896 | 9,964 | 6,376 | 9,963 | 3,874 | 9,741 | 2,219 |
|  | No. of scaffolds with i-blocks | 18 | 18 | 18 | 18 | 18 | 18 | 18 | 18 | 18 | 18 |
|  | Total length of i-blocks (Mb) | 30.63 | 28.73 | 31.37 | 28.24 | 30.07 | 24.57 | 32.29 | 16.00 | 29.54 | 9.22 |
|  | % of the genome | 15.26 | 14.31 | 15.63 | 14.07 | 15.98 | 12.24 | 16.09 | 7.97 | 14.72 | 4.59 |
| **The i-blocks contain >=3 intro sites** | No. of i-blocks | 4,805 | 5,224 | 4,880 | 5,106 | 5,007 | 4,035 | 5,004 | 2,302 | 4,883 | 1,302 |
|  | No. of scaffolds with i-blocks | 18 | 18 | 18 | 18 | 18 | 18 | 18 | 18 | 18 | 18 |
|  | Total length of i-blocks (Mb) | 23.01 | 22.94 | 23.68 | 22.46 | 24.30 | 19.48 | 24.56 | 11.65 | 21.75 | 7.41 |
|  | % of the genome | 11.47 | 11.43 | 11.80 | 11.19 | 12.11 | 9.71 | 12.24 | 5.80 | 10.84 | 3.69 |
| **The i-blocks contain >=4 intro sites** | No. of i-blocks | 2,861 | 3,661 | 2,926 | 3,628 | 3,006 | 2,868 | 3,001 | 1,562 | 2,947 | 901 |
|  | No. of scaffolds with i-blocks | 18 | 18 | 18 | 18 | 18 | 18 | 18 | 18 | 18 | 18 |
|  | Total length of i-blocks (Mb) | 18.60 | 19.00 | 19.19 | 18.55 | 19.75 | 16.53 | 19.71 | 9.43 | 17.21 | 6.03 |
|  | % of the genome | 9.27 | 9.47 | 9.56 | 9.24 | 9.84 | 8.23 | 9.82 | 4.70 | 8.58 | 3.01 |
| **The i-blocks**  **contain >=5**  **intro sites** | No. of i-blocks | 1,916 | 2,688 | 1,955 | 2,706 | 2,013 | 2,159 | 2,016 | 1,146 | 1,981 | 660 |
|  | No. of scaffolds with i-blocks | 18 | 18 | 18 | 18 | 18 | 18 | 18 | 18 | 18 | 18 |
|  | Total length of i-blocks (Mb) | 16.12 | 15.69 | 16.61 | 15.25 | 17.03 | 13.62 | 17.06 | 7.86 | 14.81 | 4.91 |
|  | % of the genome | 8.03 | 7.82 | 8.27 | 7.60 | 8.49 | 6.78 | 8.50 | 3.92 | 7.38 | 2.44 |

**Table S14 High-confidence non-introgressable j-blocks**

|  | >=1 j-sites | >=2 j-sites |
| --- | --- | --- |
| No. of j-blocks (No. scaffolds with j-blocks) | 1,189 (184) | 168 (44) |
| Length of j-blocks - Range (mean) | 3 bp - 43.51 Kb (1,010 bp) | 23 bp - 35.75 Kb (1,062 bp) |
| No. of j-sites in a block – Range (total j-sites) sites) | 1 - 6 (1,443) | 2 - 6 (422) |
| Total length of j-blocks (% of the genome) | 1,201,823 bp (0.51%) | 178,520 bp (0.075%) |
| No. of genes within j-blocks | 328 | 39 |
| No. of genes containing j-sites | 171 | 19 |

A j-block, unless explicitly stated, should have >= 2 non-introgressable sites (j-sites).

**Table S15 All functional genes within non-introgressable blocks (j-blocks) between *R. mucronata* and *R. stylosa***

| **In *Rhizophora*** | | | | **In *Arabidopsis*** [***thaliana***](javascript:;) | |
| --- | --- | --- | --- | --- | --- |
| **Gene** | **j-sites** | **L(aa)** | **sites ^a^** | **Gene** | **Function** |
| *RM_76501.12* | 2 | 1,404 | 6 | *AT4G21820* | binding / calmodulin binding protein |
| ***RM_76773.10*** | **3** | **255** | **4** | *AT2G14110* | Haloacid dehalogenase-like hydrolase (HAD) superfamily protein. Participating in pollen germination and tube growth [54]. |
| *RM_76921.24* | 2 | 539 | 0 | *AT5G04980* | DNAse I-like superfamily protein |
| *RM_76929.4* | 2 | 186 | 3 | *AT4G31940* | CYP82C4 (cytochrome P450, family 82, subfamily C, polypeptide 4). The gene encodes a cytochrome P450 enzyme, CYP82C. It is involved in [cellular response to iron ion](https://www.arabidopsis.org/servlets/TairObject?type=keyword&id=33806), [oxidation-reduction process](https://www.arabidopsis.org/servlets/TairObject?type=keyword&id=29342), [response to iron ion](https://www.arabidopsis.org/servlets/TairObject?type=keyword&id=14770),[sideretin biosynthesis](https://www.arabidopsis.org/servlets/TairObject?type=keyword&id=56071). |
| ***RM_76929.10*** | **2** | **294** | **3** | *AT1G55490* | CPN60B (chaperonin 60 beta). encodes the beta subunit of the chloroplast chaperonin 60, a homologue of bacterial GroEL. Mutants in this gene develops lesions on its leaves, expresses systemic acquired resistance (SAR) and develops accelerated cell death to heat shock stress. Other names: CPN60B, CPN60BETA1, CPNB1, LEN1. This gene can participate in embryo and seed development [57,114]. |
| *RM_76932.33* | 1 | 56 | 0 | unknown | Function unknown. |
| *RM_76963.8* | 2 | 400 | 2 | *AT1G75380* | BBD1 (bifunctional nuclease in basal defense response 1). Involved in [defense response to fungus](https://www.arabidopsis.org/servlets/TairObject?type=keyword&id=18013), [negative regulation of transcription, DNA-templated](https://www.arabidopsis.org/servlets/TairObject?type=keyword&id=12510),[protein ubiquitination](https://www.arabidopsis.org/servlets/TairObject?type=keyword&id=6911), [regulation of histone deacetylation](https://www.arabidopsis.org/servlets/TairObject?type=keyword&id=19467). Playing a role in the response to cold stress [115]. |
| ***RM_76979.9*** | **2** | **199** | **1** | *AT3G15510* | NAC2 (NAC domain containing protein 2). Involved in the regulation of [stamen development](https://www.arabidopsis.org/servlets/TairObject?type=keyword&id=31842) [55], [embryonic development](https://www.arabidopsis.org/servlets/TairObject?type=keyword&id=12744) [58] and stress response [116,117]. |
| *RM_77019.26* | 3 | 351 | 0 | *AT1G29660* | GDSL-like Lipase/Acylhydrolase superfamily protein. Enzyme group with broad substrate specificity that may catalyze acyl-transfer or hydrolase reactions with lipid and non-lipid substrates. Expressed during flower, leaf development and plant embryo globular stage. Expressed in flower, leaf, plant embryo, root, shoot and stem tissues. Participating in both early and late stages of female gametophyte development [118]. |
| *RM_77067.37* | 2 | 1,903 | 1 | *AT1G09910* | Rhamnogalacturonate lyase family protein. Also known as: F21M12.30; F21M12_30. |
| ***RM_77078.7*** | ***3*** | **1,415** | ***1*** | *AT5G11530* | EMF1 (embryonic flower 1). Involved in regulating reproductive development [52,53]. |
| *RM_77327.1* | 2 | 585 | 2 | *AT2G39090* | APC7 (tetratricopeptide repeat (TPR)-containing protein). Also known as anaphase-promoting complex 7; AtAPC7; T7F6.26; T7F6_26. Involved in [cell cycle](https://www.arabidopsis.org/servlets/TairObject?type=keyword&id=5323), [cell division](https://www.arabidopsis.org/servlets/TairObject?type=keyword&id=20664),[protein ubiquitination](https://www.arabidopsis.org/servlets/TairObject?type=keyword&id=6911). Located in nucleus. |
| ***RM_77333.68*** | **2** | **755** | **1** | *AT1G08520* | ALB1 (ALBINA 1). Encodes the CHLD subunit of the Mg-chelatase enzyme involved in chlorophyll biosynthesis. Located in chloroplast and extracellular regions. Participating in embryo and seed development [59]. Lines carrying recessive mutations of this locus are white and seedling lethal. |
| *RM_77333.219* | 2 | 167 | 0 | *AT5G52370* | 28S ribosomal S34 protein. Involved in biological process, response to cold. Located in chloroplast, mitochondrion. Expressed in [guard cell](https://www.arabidopsis.org/servlets/TairObject?type=keyword&id=19990). |
| *RM_77333.222* | 2 | 215 | 1 | *AT1G07530* | SCL14 (SCARECROW-like 14). Encodes a member of the GRAS family of transcription factors. The protein interacts with the TGA2 transcription factor and affects the transcription of stress-responsive genes [119]. The protein is found in the nucleus and is also exported to the cytoplasm. |
| *RM_77333.230* | 2 | 453 | 2 | *AT5G59380* | MBD6 (methyl-CPG-binding domain 6). Protein containing methyl-CpG-binding domain. Has sequence similarity to human MBD proteins. Involved in [gene silencing by interacting with RNA binding proteins](https://www.arabidopsis.org/servlets/TairObject?type=keyword&id=19490) [120]. |
| *RM_77333.290* | 5 | 1,056 | 1 | *AT3G45850* | P-loop containing nucleoside triphosphate hydrolases superfamily protein. FUNCTIONS IN: microtubule motor activity, ATP binding; INVOLVED IN: microtubule-based movement. |
| *RM_77333.292* | 2 | 1,398 | 2 | *AT3G45830* | nuclear factor kappa-B-binding-like protein. Function unknown. |
| ***RM_77530.24*** | **2** | **671** | **2** | *AT3G05420* | ACBP4 (acyl-CoA binding protein 4). Acyl-CoA binding protein with high affinity for oleoyl-CoA. Involved in fatty acid transport. Expressed and function in floral lipid metabolism [121]. Playing combinatory roles in pollen development [56] and distinct roles in seed development [122]. |

The six bolded genes are involved in flower development and/or gamete production and development (see also Table 2).

j-sites: the number of non-introgressable sites within the gene.

L(aa): amino acid sequence length of the gene.

**^a^** Site: No. of highly differentiated amino acids between *R. mucronata* and *R. stylosa* (see also Fig. S16).

**Supplementary Figures:**


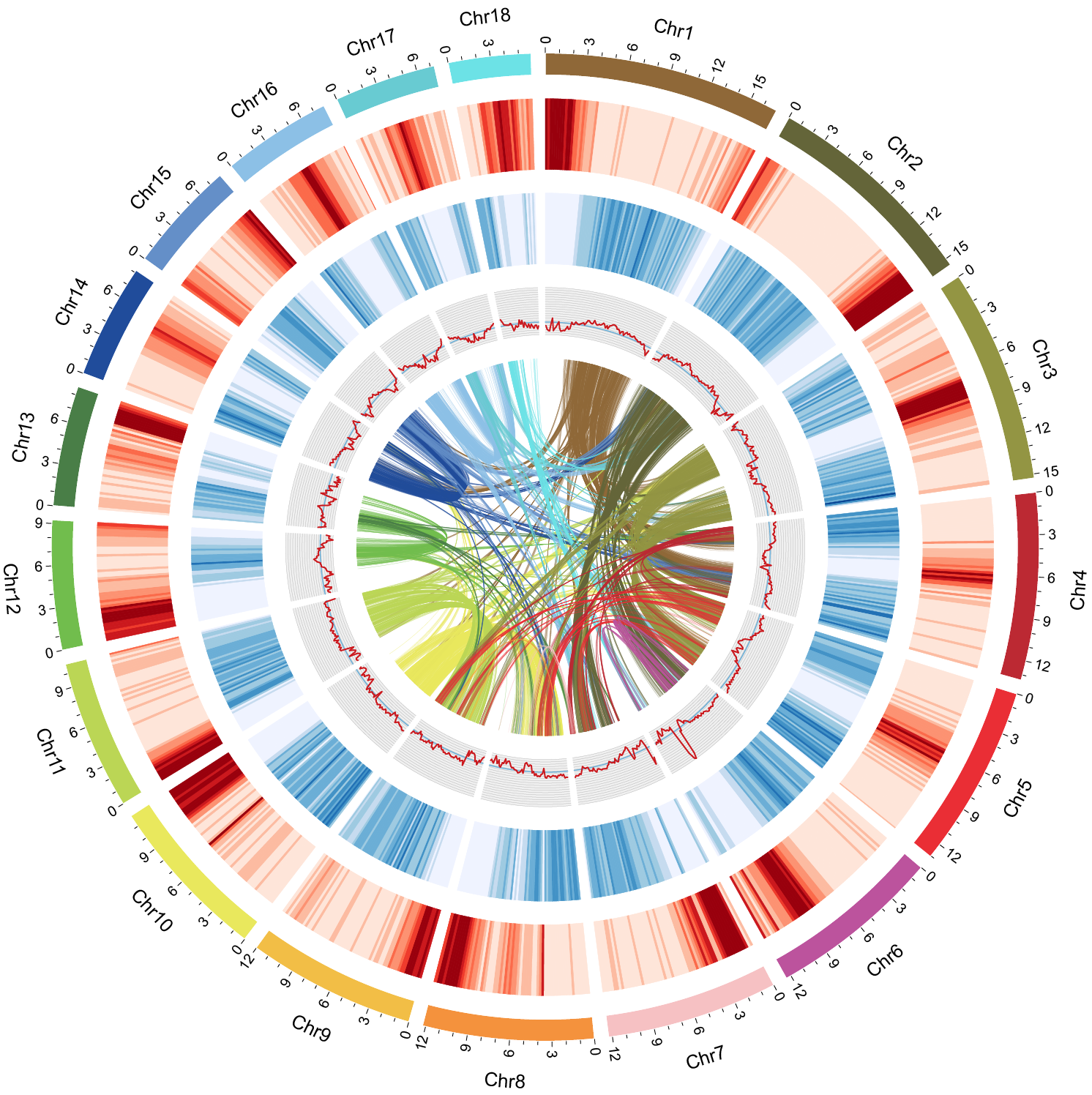


**Figure S1. Features of the *R. mucronata* genome*.*** Circular tracks represent, from outer to inner, top 18 longest scaffolds (Chr1-Chr18, with length >= 5Mb), percentage of repeats (5.73-99.92%, the darker the higher), gene density (0-47, the darker the higher), GC content (29.73-51.97%) with mean value 35.69 (blue line), and the spectrum of collinear analysis (each line connects one pair of homologous genes and a cluster of such lines represents one collinear block). All statistics are calculated in 200 Kb windows.


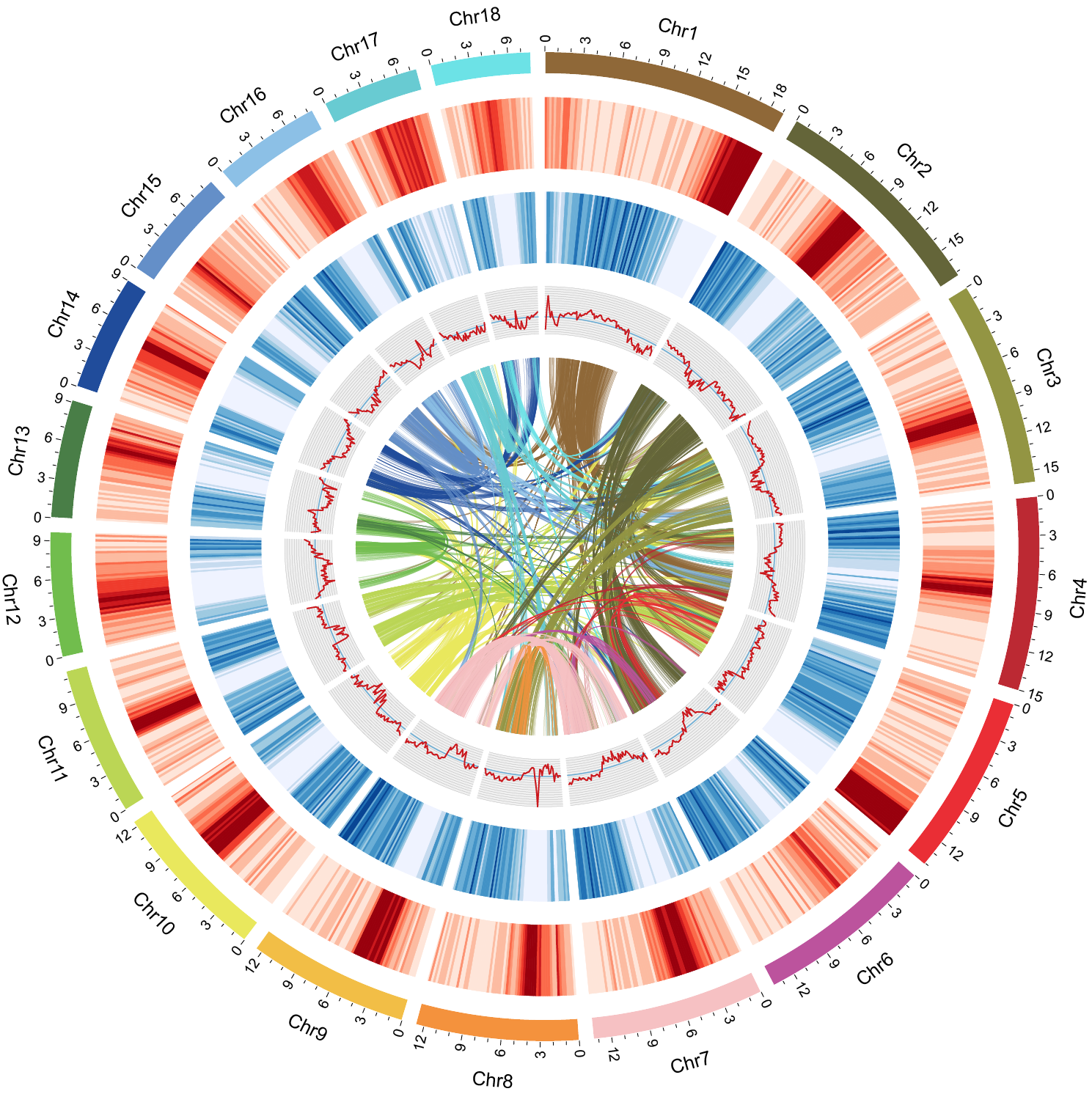


**Figure S2. Features of the *R. stylosa* genome*.*** Circular tracks represent, from outer to inner, top 18 longest scaffolds (Chr1-Chr18), percentage of repeats (3.97-99.7%, the darker the higher), gene density (0-42, the darker the higher), GC content (29.75-46.45%) with mean value 35.78 (blue line), and the spectrum of collinear analysis (each line connects one pair of homologous genes and a cluster of such lines represents one collinear block). All statistics are calculated in 200 Kb windows.


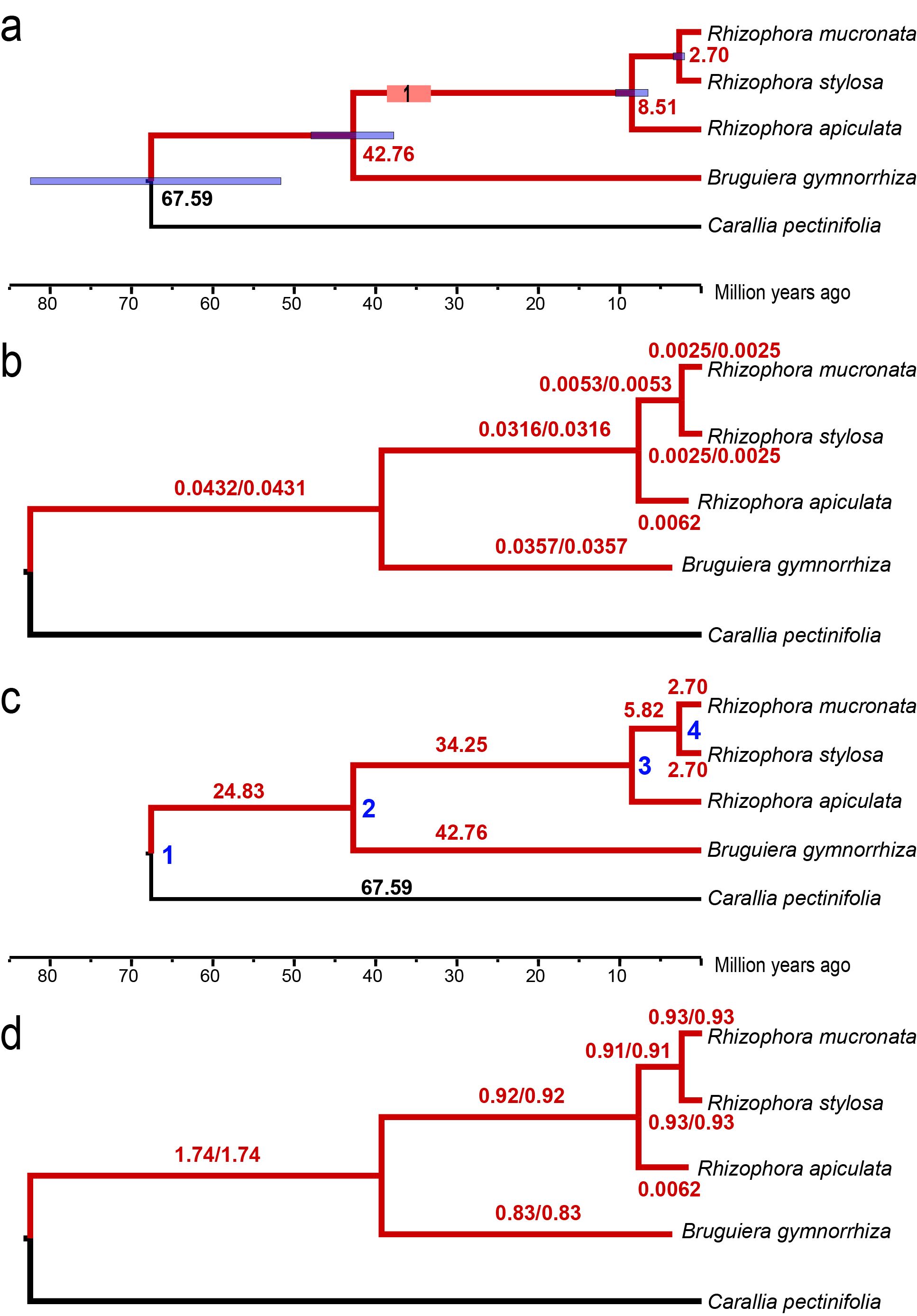


**Figure S3. Phylogenetic relationships and evolution of Rhizophoraceae species.** (a) Phylogenetic relationships and divergence time estimation of five species in the Rhizophoraceae family. The blue bars show 95% credible intervals of divergence time for each node. Red rectangle with number represents the earliest known fossil record of mangrove lineages (see the above supplementary methods). (b) The phylogenetic tree. The paired numbers above each branch represents branch lengths generated by RAXML (first) and IQTREE (second) using the model GTR+gamma and 1000 bootstrap replicates. All nodes are 100% supported. (c) A Phylogenetic tree showing node numbers and time span of each branch. The nodes are numbered (1-4 in blue) in order to show the divergence time, corresponding to that in the Table S3. The time span is above/below each branch, with time unit one million years. (d) Substitution rates (x10^-9^ per site per year) estimated for each branch. The substitution rates are estimated through dividing branch length by the time span of the branch. In all figures, branches in red represent mangrove species.


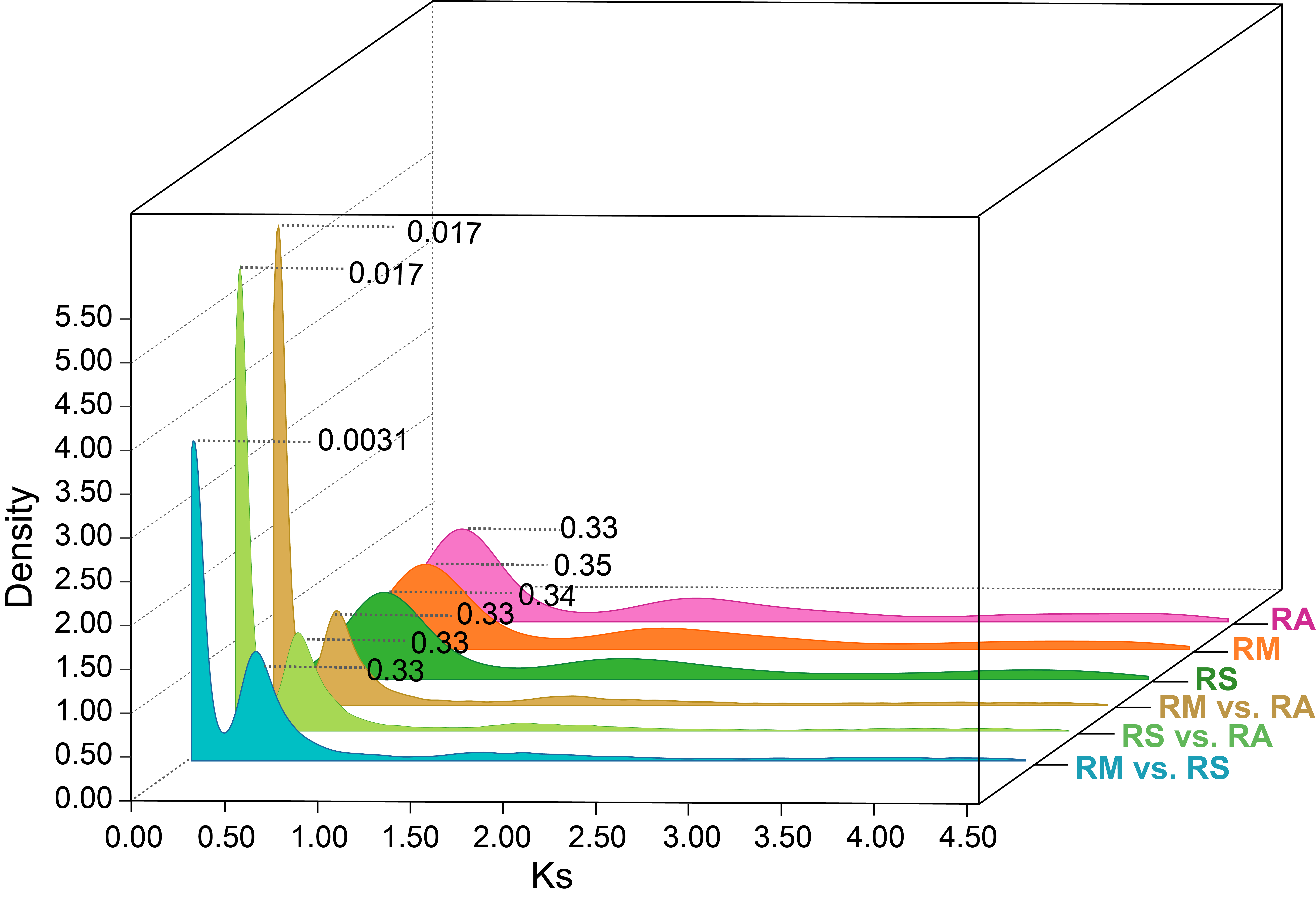


**Figure S4. Intra and inter-specific Ks distributions of three *Rhizophora* species.** Ks values at peaks are next to dotted lines. RM (orange): *R. mucronata*. RS (green): *R. stylosa*. RA (purple): *R. apiculata*. Inter-specific Ks distributions include RM vs. RS (blue), RM vs. RA (light green) and RS vs. RA (dark yellow). The Ks was calculated using KaKs_Calculator 2.0 (with the model YN-HKY) [97].


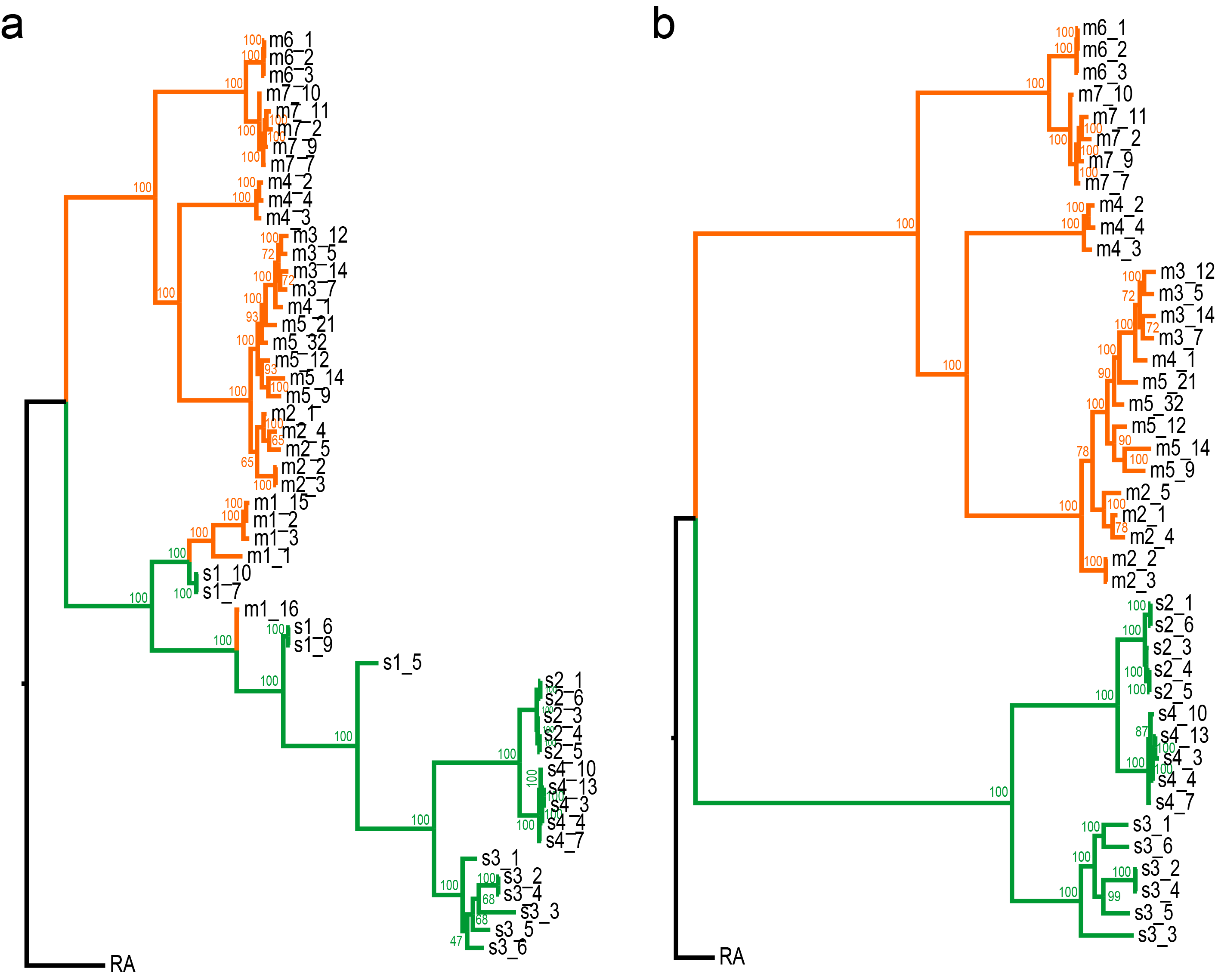


**Figure S5. Phylogenetic relationships of *R. mucronata* and *R. stylosa* samples with (a) or without (b) sympatric populations m1 and s**1. Branches of *R. mucronata* populations (or individuals) are colored in orange while those of *R. stylosa* are in green. The Maximum Likelihood (ML) trees were generated by IQTREE [38] with 100 bootstrap replicates. Bootstrap values (supporting rate %) are provided close to each node.


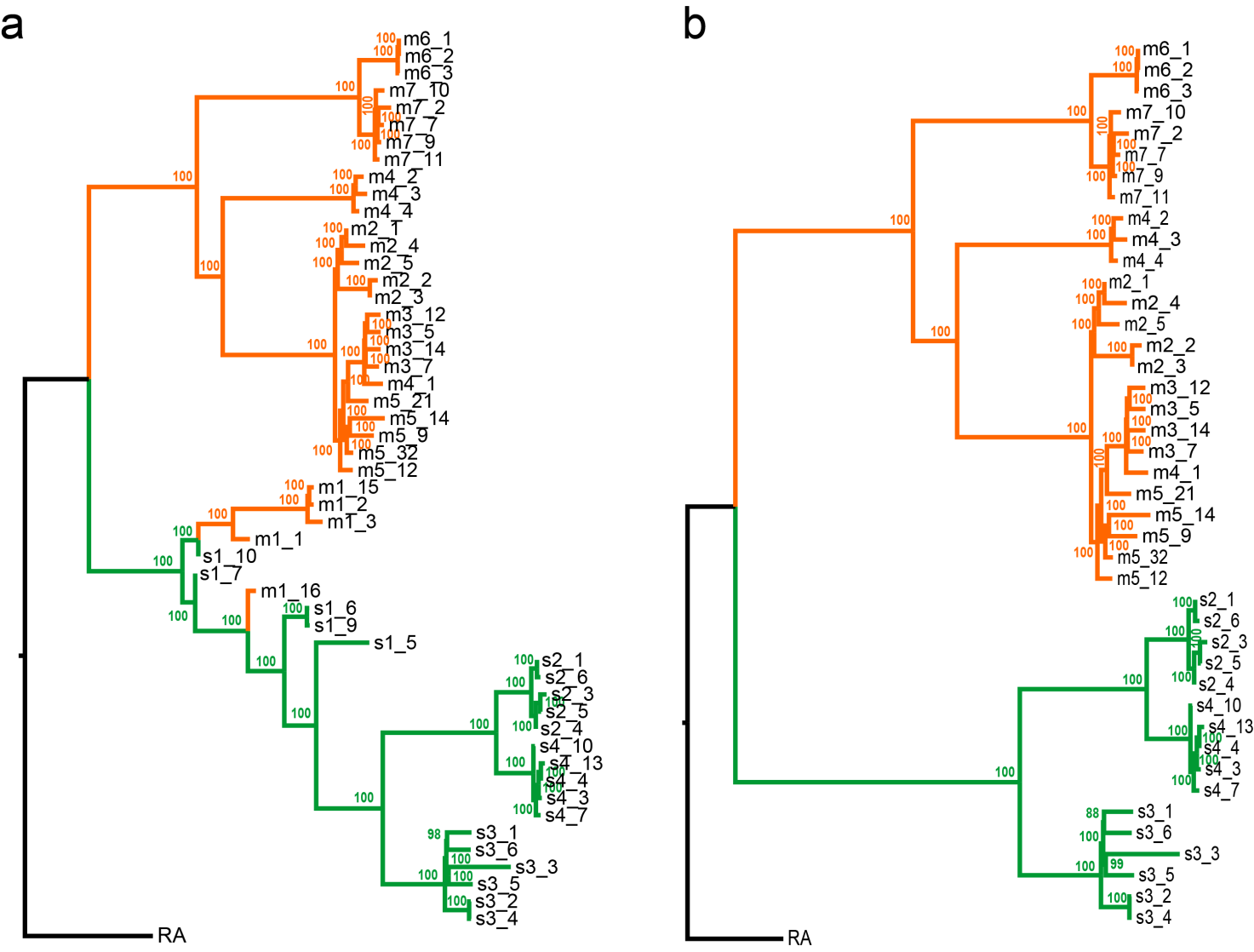


**Figure S6. Phylogenetic relationships of *R. mucronata* and *R. stylosa* samples with (a) or without (b) sympatric populations m1 and s1.** Branches of *R. mucronata* populations (or individuals) are colored in orange while those of *R. stylosa* are in green. The Neighbor-joining (NJ) trees were generated by MEGA7 [39] with 100 bootstrap. Bootstrap values (supporting rate %) are provided close to each node.


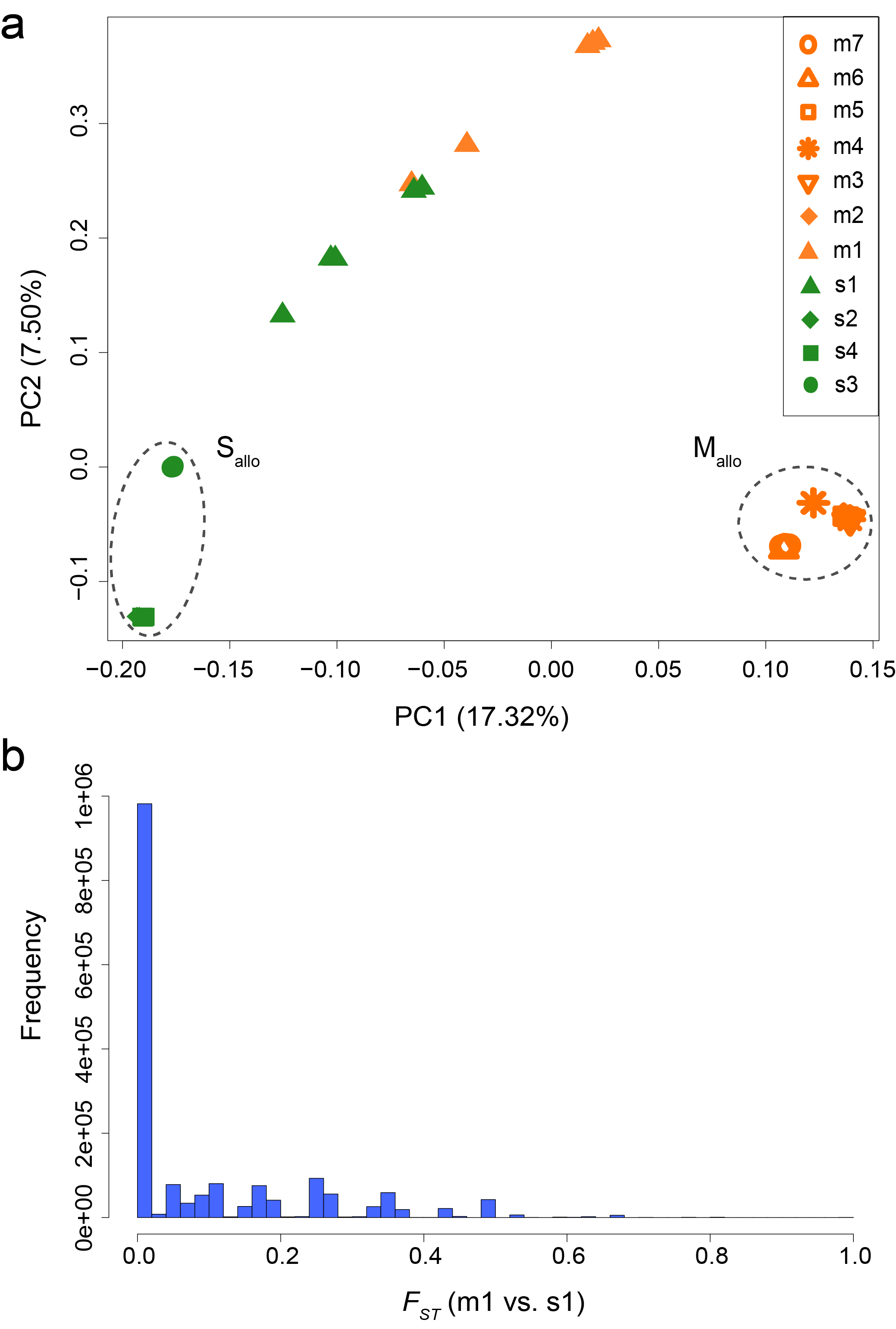


**Figure S7. (a) PCA plot of all populations** [40]**.** *R. mucronata* individuals are colored in orange while *R. stylosa* individuals in green. Allopatric populations (M_allo_ and S_allo_) are highlighted by dotted lines. M_allo_ contains populations m2-m7; S_allo_ includes populations s2-s4. (b) The spectrum of the FST statistic between the m1 and s1 (or DR) samples.


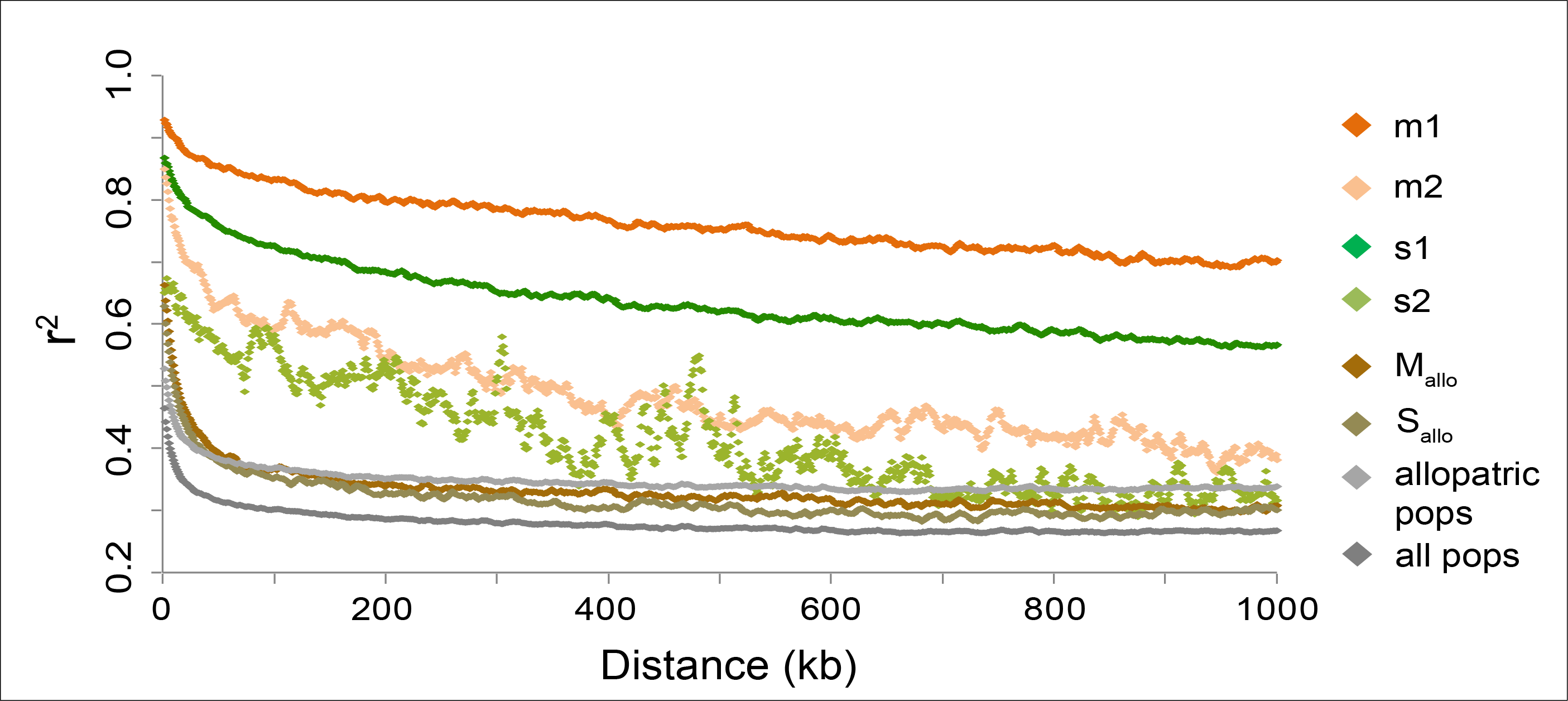


**Figure S8. Decay of linkage disequilibrium in *R. mucronata* and *R. stylosa* populations measured by r^2^.** M_allo_ contains populations m2-m7; S_allo_ includes populations s2-s4; “allopatric pops” represents all allopatric populations m2-m7 and s2-s4; “all pops” are values for all populations m1-m7 and s1-s4 together.


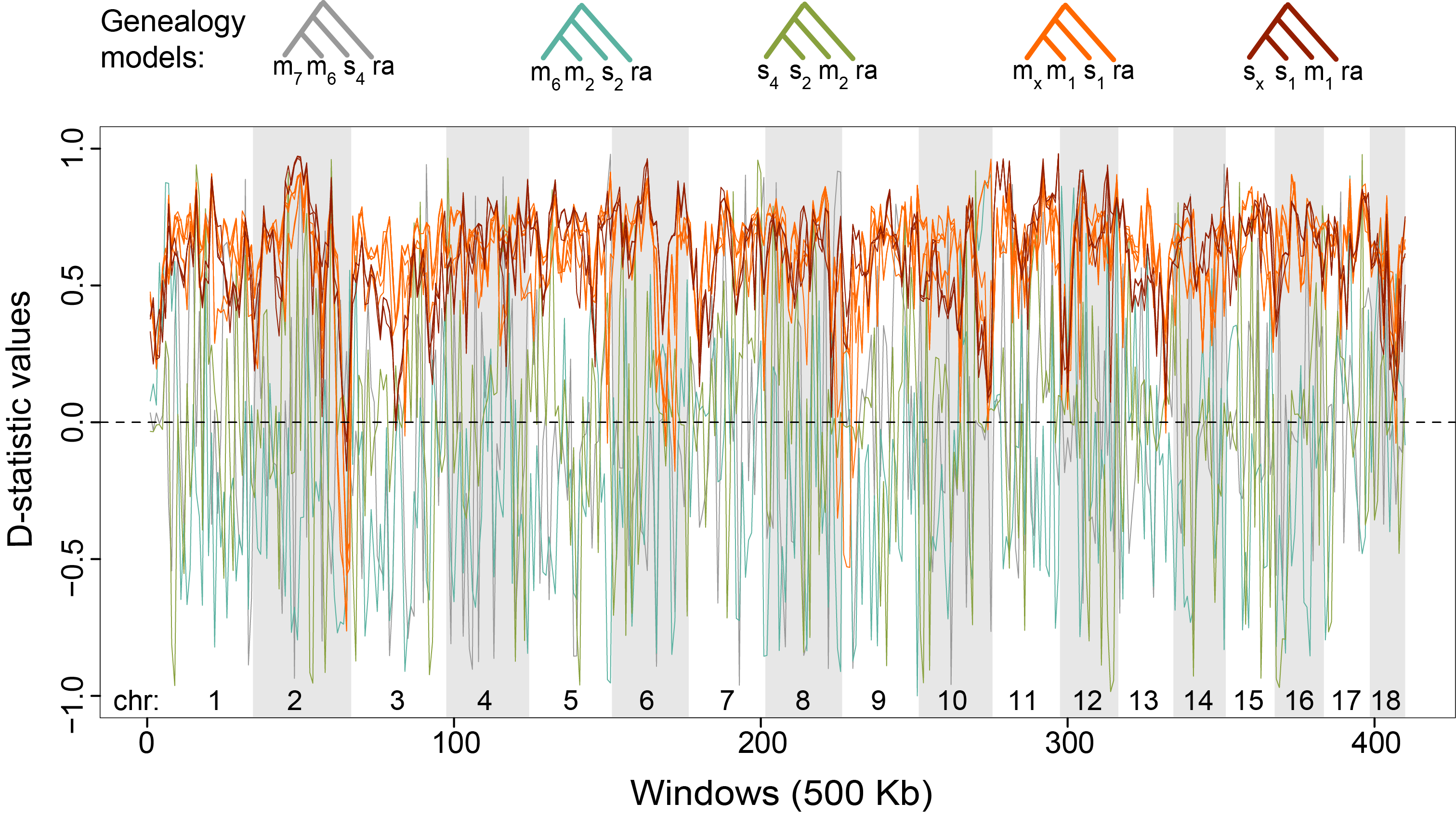


**Figure S9. Patterson's *D* statistic scan across the genome for the genealogy models above, showing genome wide evidence of gene flow between sympatric populations m1 and s1.** The curves are colored by model type. In the last two genealogy models, m**_x_** represents *R. mucronata* population m2, m3, m4, m5, m6 or m7, and s**_x_** represents *R. stylosa* population s2, s3 or s4 (see Table S10 for detail information). The top 18 longest scaffolds (chr1-18) are shown and sibling scaffolds are distinguished by gray shadows.


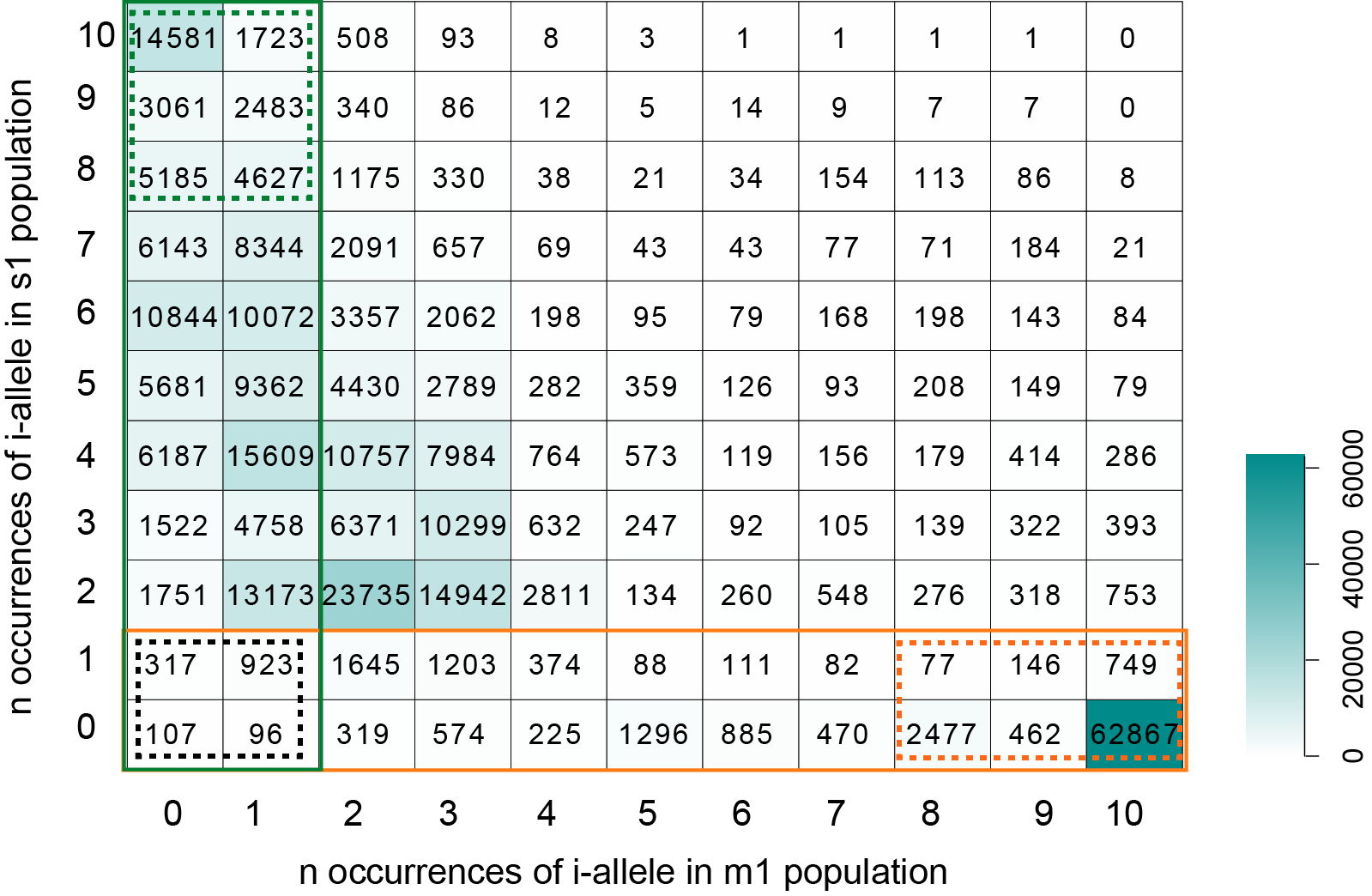


**Figure S10. Distribution of i-allele occurrences in m1 (orange) and s1 (green) populations.** Given the five individuals (or 10 haploid genomes) from each population, the occurrence ranges from 0 to 10. The actual numbers of sites are shown. Sites in the orange (in m1) and green (in s1) solid boxes correspond to the site distributions in Fig. 4B. The orange and green dotted boxes contain the i-sites (with >=8 occurrences of i-allele) in m1 and s1 populations, respectively. The black dotted box shows non-introgressable sites (j-sites) with <=1 occurrences of i-allele both in m1 and s1 samples.


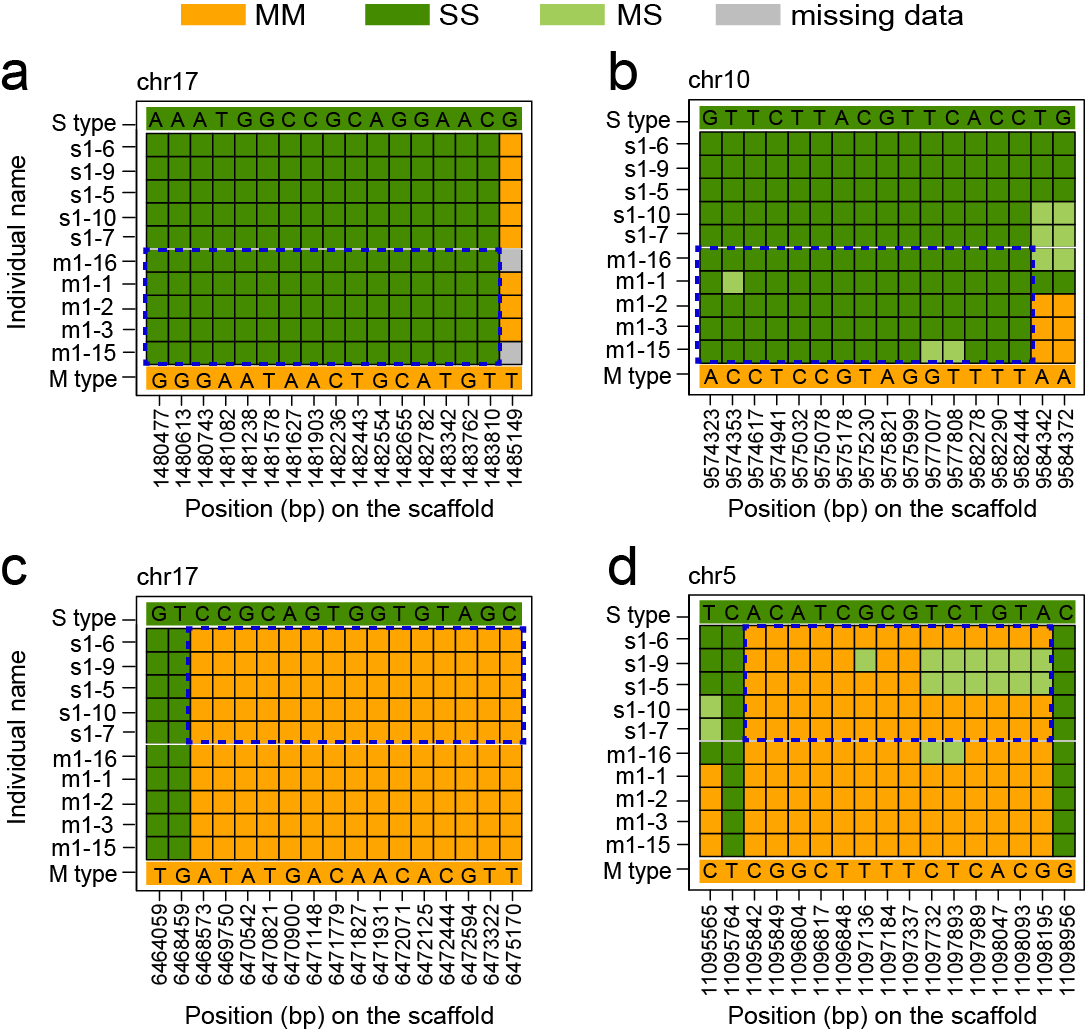


**Figure S11. Examples of i-blocks (in blue dotted boxes) in m1 genomes (a and b) and in s1 genomes (c and d) at the site level.** To make the figures more intuitive and concise, only i-sites and d-sites (*F_ST_* > 0.8 between M_allo_ and S_allo_) are shown. Note that i-sites also belong to d-sites (see the text). The top (S type) and bottom (M type) rows in all figures show the dominant bases in S_allo_ and M_allo_, respectively. Each retained row indicates an individual with one vertical line indicating a site. All 10 individuals from the sympatric s1 and m1 populations are shown. Each site is color coded for its genotype: MM (orange), MS (light green) and SS (green) type, where M is for *R. mucronata* and S for *R. stylosa* (see Materials and Methods, and Supplementary Methods).


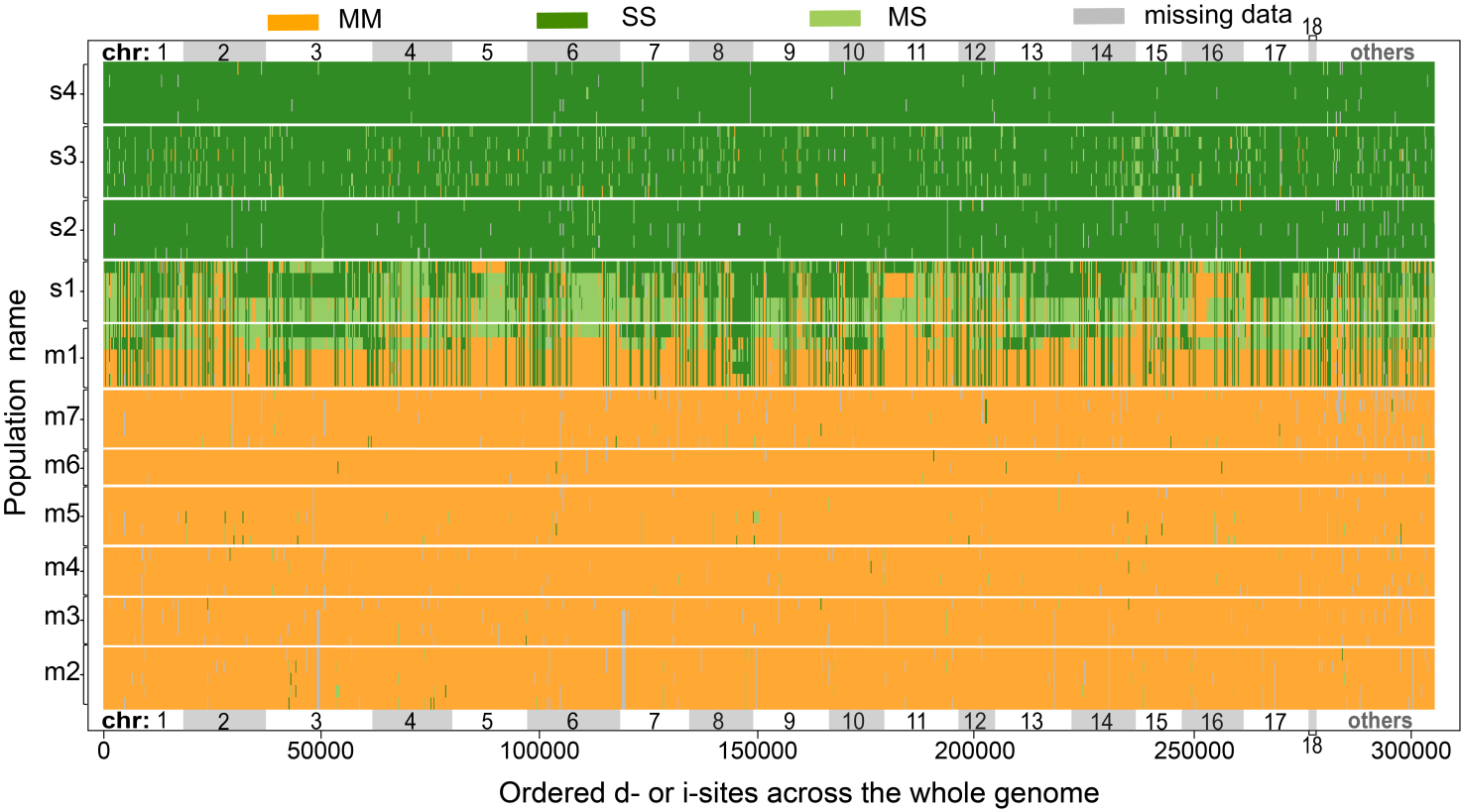


**Figure S12. The genome-wide landscape of i-blocks. All 52 *R. mucronata* and *R. stylosa* individuals are shown.** Top 18 longest scaffolds (chr1-18) and the rest of the genome (others) are marked and sibling scaffolds are distinguished by gray rectangles. In each ideogram, all d- and i-sites are displayed consecutively. Each site is color-coded for the MM, MS, and SS genotypes as in Fig. S11.


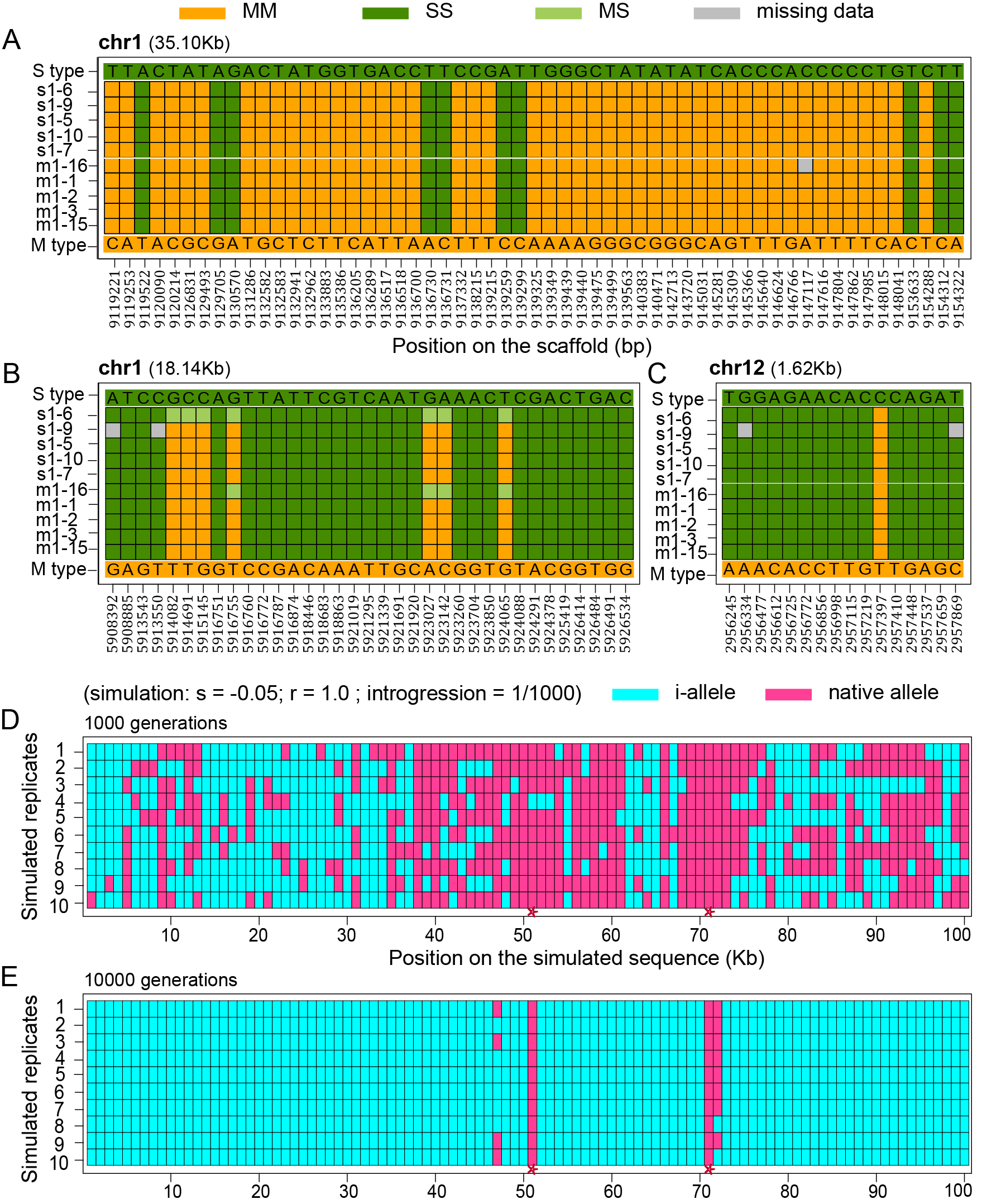


**Figure S13. Examples of i-blocks and simulated introgressions in haploid 100 Kb genomes.** (A-C) Examples of i-blocks in m1 and s1 samples at the site level that show the fine-scale delineation. Color codes are the same as in Fig. S11. (D-E) Simulated introgressions in haploid 100 Kb genomes. This example is from a simulation with strong selection (s = -0.05), high recombination (r = 1.0 for per 100Kb per generation), and low introgression (1/1000 per generation). Two time points are given (see Materials and Methods, Supplementary Methods and Fig. S14 for details). Two speciation genes (or loci) under selection at 51 and 71 Kb are marked by red stars at the bottom. Introgressed and non-introgressed sites are marked in blue and pink, respectively. Note that very fine delineations of blocks are possible under the simulated conditions.


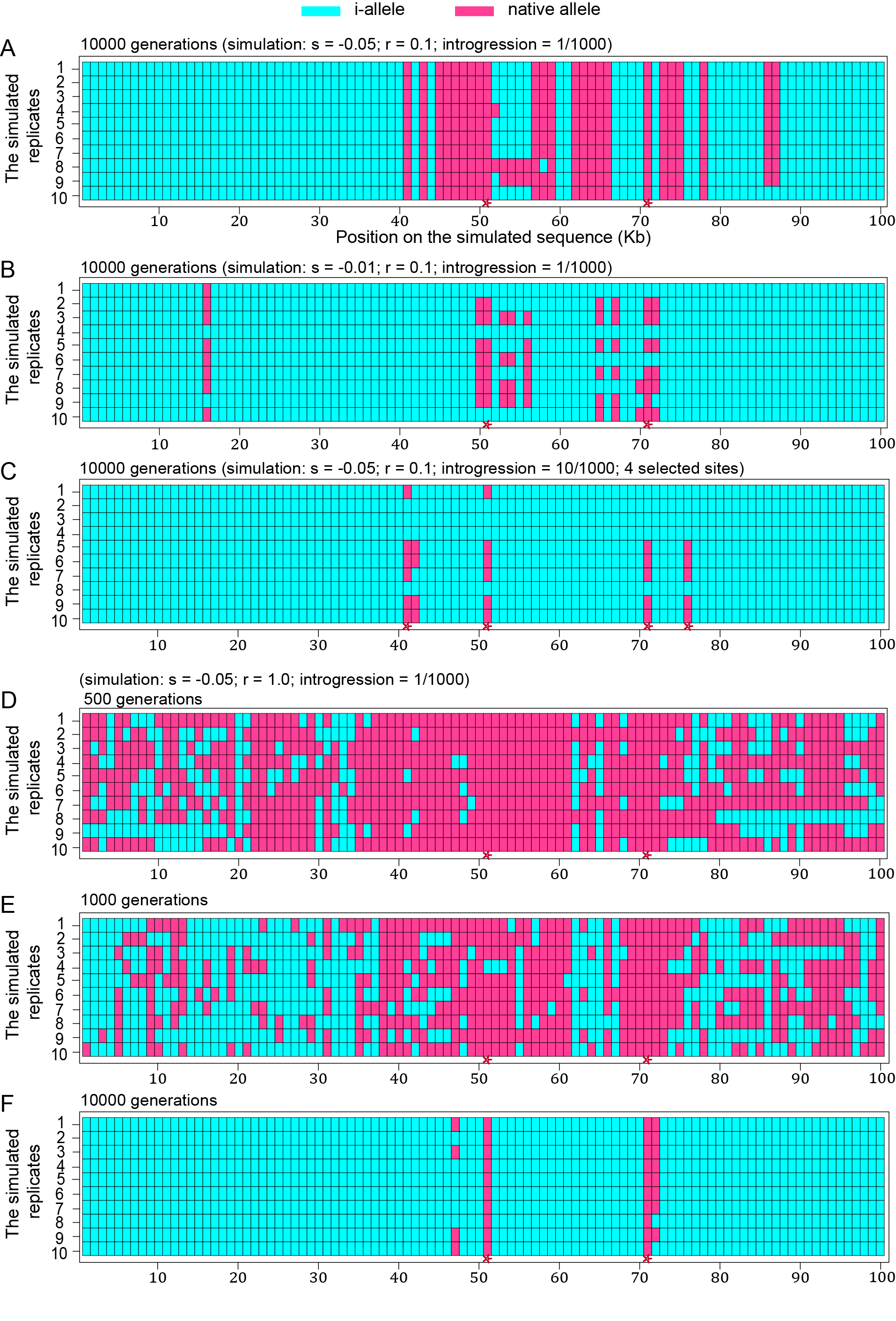


**Figure S14. Simulated introgressions in haploid 100 Kb genomes. Speciation genes (or loci) are marked by red stars at the bottom. Introgressed and non-introgressed (or native) sites are marked in blue and pink, respectively.** (A) Simulated results of 10000 generations under strong selection (s = -0.05), low recombination rate (r = 0.1 for per 100 Kb per generation), and low introgression (m=0.001 per generation). Native alleles are not purified at neutral loci. (B) Simulated results of 10000 generations under weak selection (s = -0.01), low recombination rate (r = 0.1 for per 100 Kb per generation), and low introgression (m=0.001 per generation). Speciation loci under selection also show introgressions. (C) Simulated results of 10000 generations under strong selection (s = -0.05) plus four loci under selection (#41, #51, #71 and #76), low recombination rate (r = 0.1 for per 100 Kb per generation), and high introgression (m=0.01 per generation). Speciation loci under selection have shown introgressions as well. (D-F) Simulated results under strong selection (s = -0.05), high recombination (r = 1.0 for per 100 Kb per generation), and low introgression (m=0.001 per generation). Three time points are given. This is closest to the expected pattern.


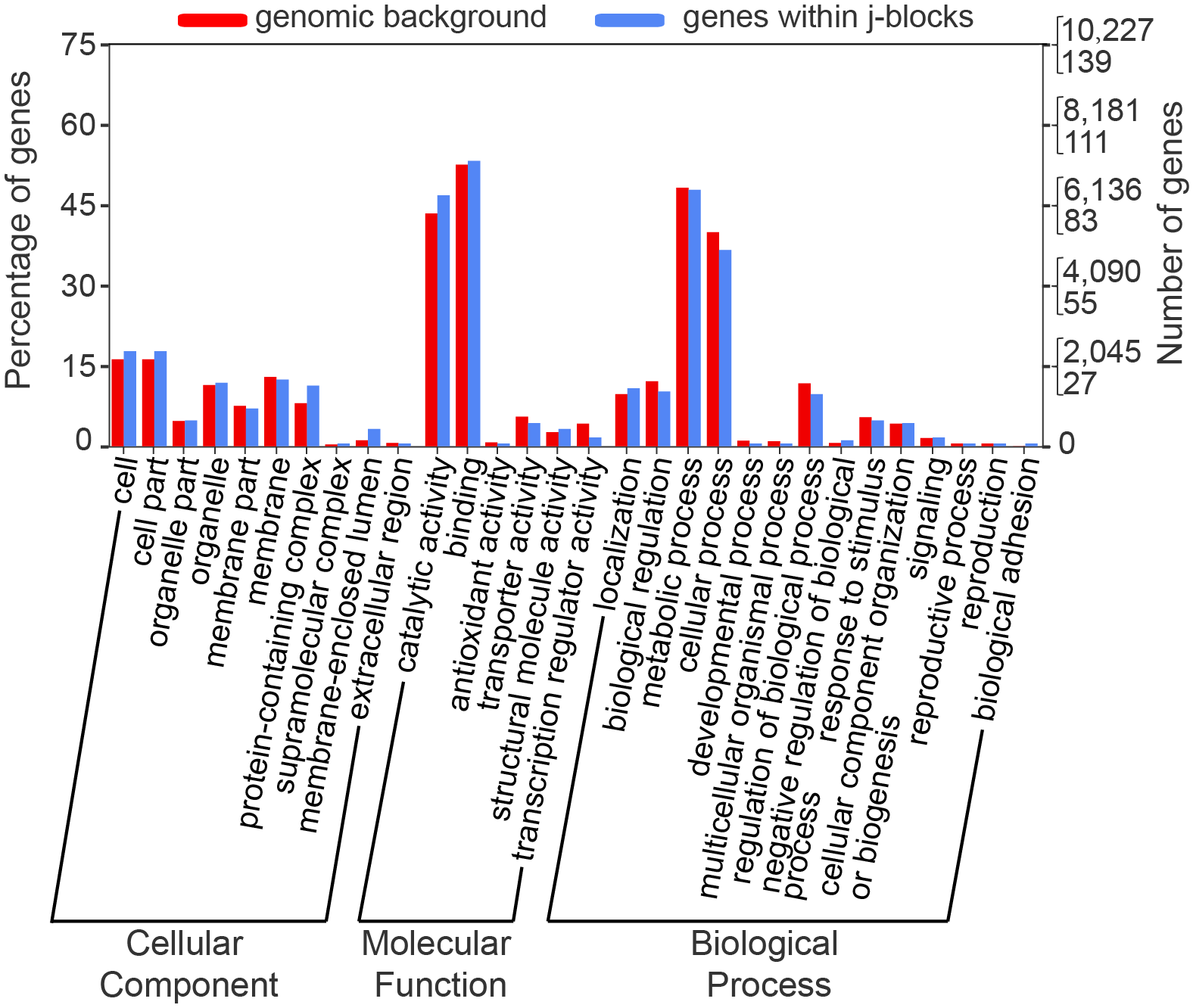


**Figure S15. GO term (level 2) distribution for all genes (328) in the j-blocks (or non-introgressable blocks) between *R. mucronata* and *R. stylosa*.** We use genes from the whole genome as genetic background. In total, 186 genes were assigned to at least one GO term and grouped into three main GO categories and 30 GO terms. WEGO 2.0 (Web Gene Ontology Annotation Plot, <http://wego.genomics.org.cn/>) was used for this analysis [123].


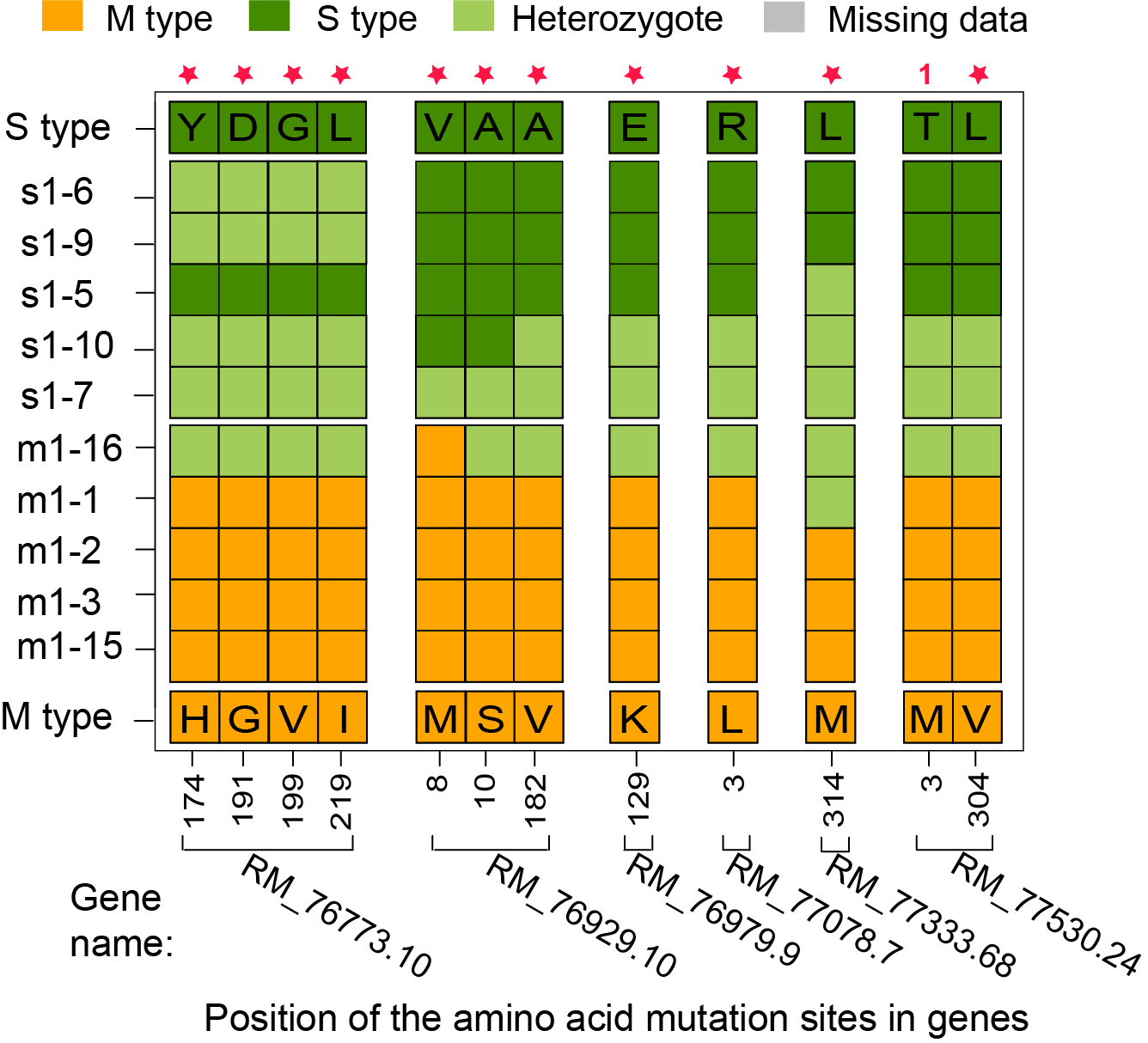


**Figure S16. Highly differentiated amino acids between *R. mucronata* and *R. stylosa* samples in the six genes involved in flower development.** Sites marked by red stars are fixed between allopatric *R. mucronata* (m2-m7) and *R. stylosa* (s2-s4) samples. Only one site contains one (number in red) heterozygote in the s3 (*R. stylosa*) population. Each site is color-coded for the M type (orange), Heterozygote (light green), and S type (green) (M is for allopatric *R. mucronata* dominant amino acid and S for allopatric *R. stylosa* dominant amino acid).

**Supplementary references**

76. Duke NC. Mangrove floristics and biogeography revisited: Further deductions from biodiversity hot spots, ancestral discontinuities, and common evolutionary processes. *Mangrove Ecosystems: A Global Biogeographic Perspective: Structure, Function, and Services*. 2017, Pp.17-53.

77. Weisenfeld NI, Kumar V, Shah P *et al.* Direct determination of diploid genome sequences. *Genome Res* 2017;**27**:757–67.

78. Tyagi AP. Cytogenetics and reproductive biology of mangroves in Rhizophoraceae. *Aust J Bot* 2002;**50**:601–5.

79. Subramanian D. Cytological Studies of some Mangroove Flora of Tamilnadu. *Cytologia (Tokyo)* 1988;**53**:87–92.

80. Tarailo-Graovac M, Chen N. Using RepeatMasker to identify repetitive elements in genomic sequences. *Curr Protoc Bioinforma* 2009;**25**:4.10.1-4.10.14.

81. Bao W, Kojima KK, Kohany O. Repbase Update, a database of repetitive elements in eukaryotic genomes. *Mob DNA* 2015;**6**:11.

82. Flynn JM, Hubley R, Goubert C *et al.* RepeatModeler2 for automated genomic discovery of transposable element families. *Proc Natl Acad Sci U S A* 2020;**117**:9451–7.

83. Xu Z, Wang H. LTR-FINDER: An efficient tool for the prediction of full-length LTR retrotransposons. *Nucleic Acids Res* 2007;**35**:W265--W268.

84. Benson G. Tandem repeats finder: A program to analyze DNA sequences. *Nucleic Acids Res* 1999;**27**:573–80.

85. Kent WJ. BLAT - The BLAST-like alignment tool. *Genome Res* 2002;**12**:656–64.

86. Birney E, Clamp M, Durbin R. GeneWise and Genomewise. *Genome Res* 2004;**14**:988–95.

87. Kim D, Paggi JM, Park C *et al.* Graph-based genome alignment and genotyping with HISAT2 and HISAT-genotype. *Nat Biotechnol* 2019;**37**:907–15.

88. Pertea M, Pertea GM, Antonescu CM *et al.* StringTie enables improved reconstruction of a transcriptome from RNA-seq reads. *Nat Biotechnol* 2015;**33**:290–5.

89. Stanke M, Keller O, Gunduz I *et al.* AUGUSTUS: A b initio prediction of alternative transcripts. *Nucleic Acids Res* 2006;**34**:W435–9.

90. Burge C, Karlin S. Prediction of complete gene structures in human genomic DNA. *J Mol Biol* 1997;**268**:78–94.

91. Majoros WH, Pertea M, Salzberg SL. TigrScan and GlimmerHMM: Two open source ab initio eukaryotic gene-finders. *Bioinformatics* 2004;**20**:2878–9.

92. Haas BJ, Salzberg SL, Zhu W *et al.* Automated eukaryotic gene structure annotation using EVidenceModeler and the Program to Assemble Spliced Alignments. *Genome Biol* 2008;**9**:R7.

93. Li H. Aligning sequence reads, clone sequences and assembly contigs with BWA-MEM. *arXiv: 13033997v2[q-bioGN]* 2013, DOI: arXiv:1303.3997.

94. Wang GD, Shao XJ, Bai B *et al.* Structural variation during dog domestication: Insights from gray wolf and dhole genomes. *Natl Sci Rev* 2019;**6**:110–22.

95. Wang Y, Tang H, Debarry JD *et al.* MCScanX: A toolkit for detection and evolutionary analysis of gene synteny and collinearity. *Nucleic Acids Res* 2012;**40**:e49.

96. Krzywinski M, Schein J, Birol I *et al.* Circos: An information aesthetic for comparative genomics. *Genome Res* 2009;**19**:1639–45.

97. Wang D, Zhang Y, Zhang Z *et al.* KaKs_Calculator 2.0: A Toolkit Incorporating Gamma-Series Methods and Sliding Window Strategies. *Genomics, Proteomics Bioinforma* 2010;**8**:77–80.

98. Gillespie JH. *Population Genetics*. The Johns Hopkins University Press, 1998.

99. Emms DM, Kelly S. OrthoFinder: solving fundamental biases in whole genome comparisons dramatically improves orthogroup inference accuracy. *Genome Biol* 2015;**16**:157.

100. Lin T, Xu X, Ruan J *et al.* Genome analysis of Taraxacum kok-saghyz Rodin provides new insights into rubber biosynthesis. *Natl Sci Rev* 2018;**5**:78–87.

101. Buchfink B, Xie C, Huson DH. Fast and sensitive protein alignment using DIAMOND. *Nat Methods* 2015;**12**:59–60.

102. Edgar RC. MUSCLE: A multiple sequence alignment method with reduced time and space complexity. *BMC Bioinformatics* 2004;**5**:113.

103. Suyama M, Torrents D, Bork P. PAL2NAL: Robust conversion of protein sequence alignments into the corresponding codon alignments. *Nucleic Acids Res* 2006;**34**:W609–12.

104. Darriba D, Taboada GL, Doallo R *et al.* JModelTest 2: More models, new heuristics and parallel computing. *Nat Methods* 2012;**9**:772.

105. Yang Z. PAML 4: Phylogenetic analysis by maximum likelihood. *Mol Biol Evol* 2007;**24**:1586–91.

106. Graham A. Paleobotanical Evidence and Molecular Data in Reconstructing the Historical Phytogeography of Rhizophoraceae 1 . *Ann Missouri Bot Gard* 2006;**93**:325–34.

107. Collinson ME. *Fossil Plants of the London Clay*. Palaeontological Association Field Guide to Fossils, 1983.

108. Muller J. Fossil pollen records of extant angiosperms. *Bot Rev* 1981;**47**:1–142.

109. Li H, Handsaker B, Wysoker A *et al.* The Sequence Alignment/Map format and SAMtools. *Bioinformatics* 2009;**25**:2078–9.

110. McKenna A, Hanna M, Banks E *et al.* The Genome Analysis Toolkit: a MapReduce framework for analyzing next-generation DNA sequencing data. *Genome Res* 2010;**20**:1297–303.

111. Nei M. Analysis of gene diversity in subdivided populations. *Proc Natl Acad Sci U S A* 1973;**70**:3321–3.

112. Nei M, Li WH. Mathematical model for studying genetic variation in terms of restriction endonucleases. *Proc Natl Acad Sci U S A* 1979;**76**:5269–73.

113. Wright S. The genetic structure of populations. *Ann Eugenetics* 1951;**16**:97–159.

114. Hajduch M, Hearne LB, Miernyk JA *et al.* Systems Analysis of Seed Filling in Arabidopsis: Using General Linear Modeling to Assess Concordance of Transcript and Protein Expression. *Plant Physiol* 2010;**152**:2078–87.

115. Sharma N, Cram D, Huebert T *et al.* Exploiting the wild crucifer Thlaspi arvense to identify conserved and novel genes expressed during a plant’s response to cold stress. *Plant Mol Biol* 2007;**63**:171–84.

116. Aslam M, Grover A, Sinha VB *et al.* Isolation and characterization of cold responsive NAC gene from Lepidium latifolium. *Mol Biol Rep* 2012;**39**:9629–38.

117. Chen T, Liu J, Lei G *et al.* Effects of tobacco ethylene receptor mutations on receptor kinase activity, plant growth and stress responses. *Plant Cell Physiol* 2009;**50**:1636–50.

118. Yu HJ, Hogan P, Sundaresan V. Analysis of the female gametophyte transcriptome of Arabidopsis by comparative expression profiling. *Plant Physiol* 2005;**139**:1853–69.

119. Fode B, Siemsen T, Thurow C *et al.* The arabidopsis GRAS protein SCL14 interacts with class II TGA transcription factors and is essential for the activation of stress-inducible promoters. *Plant Cell* 2008;**20**:3122–35.

120. Parida AP, Sharma A, Sharma AK. AtMBD6, a methyl CpG binding domain protein, maintains gene silencing in Arabidopsis by interacting with RNA binding proteins. *J Biosci* 2017;**42**:57–68.

121. Ye ZW, Xu J, Shi J *et al.* Kelch-motif containing acyl-CoA binding proteins AtACBP4 and AtACBP5 are differentially expressed and function in floral lipid metabolism. *Plant Mol Biol* 2017;**93**:209–25.

122. Hsiao AS, Haslam RP, Michaelson L V. *et al.* Arabidopsis cytosolic acyl-CoA-binding proteins ACBP4, ACBP5 and ACBP6 have overlapping but distinct roles in seed development. *Biosci Rep* 2014;**34**:865–77.

123. Ye J, Zhang Y, Cui H *et al.* WEGO 2.0: A web tool for analyzing and plotting GO annotations, 2018 update. *Nucleic Acids Res* 2018;**46**:W71–5.
